# Deciphering the miRNA transcriptome of granulosa cells from dominant and subordinate follicles at first follicular wave in goat

**DOI:** 10.1080/10495398.2023.2259967

**Published:** 2023-09-26

**Authors:** Jinzhu Meng, Yuanyuan Zhao, Xingchao Song, Qingming An, Zhenyang Wu

**Affiliations:** a Key Laboratory for Biodiversity Conservation and Utilization in the Fanjing Mountain Region, Tongren University, Tongren, P.R. China; b College of Veterinary Medicine, Hunan Agricultural University, Changsha, P.R. China

**Keywords:** Goat, follicle development, small RNA sequencing, miRNA, target gene

## Abstract

In goats, most follicles in the ovaries will be atresia and only a few dominant follicles (DFs) may eventually mature and ovulate at a follicular wave. To investigate the potential microRNAs (miRNAs) that regulate the expression of genes associated with follicular dominance or atresia, small RNA sequencing was performed on granulosa cells of DF and subordinate follicle at the first follicular wave in goats. A total of 108 differentially expressed miRNAs were detected in the two types of follicle granulosa cells: 16 upregulated miRNAs and 92 downregulated miRNAs. Kyoto Encyclopedia of Genes and Genomes analysis of the target genes showed that *TKTL1*, *LOC102187810*, *LOC102184409* and *ALDOA* are closely associated with follicle dominance and are involved in the pentose phosphate pathway. Furthermore, a coexpression network of miRNAs and follicular dominance-related genes was constructed. The qPCR results well correlated with the small RNA sequencing data. Our findings provide new insight for exploring the molecular mechanism of miRNAs in regulating follicular development in goats.

## Introduction

In goats, follicles develop in the form of a wave-like pattern,^1^^,^^2^ there are between two and six follicle development waves during oestrous cycles, with three to four waves being the most prevalent.^3^ Each follicle development wave is preceded by a transient rise of follicle-stimulating hormone concentration in serum, and a cluster of antral follicles are induced to recruit, of which a specific number will be selected to become dominant follicles (DFs), while the remaining follicles known as subordinate follicles (SFs) begin to undergo atresia.^4^^,^^5^ As a component of the follicle, granulosa cells offer a stable internal environment for oocyte development during follicular dominance.^6^ There have been many studies on the hormones and factors involved in follicular dominance. Cytochrome P450 side-chain cleavage (P450scc) and cytochrome P450 aromatase (P450arom) participate in negative feedback regulation and promote follicular dominance through oestradiol (E_2_) secretion by granulosa cells.^7^ The insulin-like growth factor (IGF) can significantly promote E_2_ secretion by granulosa cells, accelerating follicular selection and dominance.^8^ Yet, in the absence of ovulatory signals, the DF also undergoes atresia and the second follicular development wave is initiated. However, the molecular mechanisms governing the wave-like pattern of follicular development are incompletely described.

MicroRNAs (miRNAs) are endogenous noncoding single-stranded RNA molecules with a length of 18–24 nt that are involved in post-transcriptional regulation of gene expression in animals and plants.^9^ In the previous study, nearly all the mature sequences coding mRNA transcripts contain miRNA response elements, which implies miRNAs play a crucial role in developmental, physiological and pathological processes.^10^ Moreover, one miRNA can regulate multiple genes, and conversely, a single gene can be regulated by more than one miRNA.^11^ In recent years, the emergence of miRNA as a breakthrough point in studying animal reproductive performance has provided a new perspective for understanding the mechanism of follicle development. Let-7e regulates granulosa cell proliferation and autophagy by inhibiting the p21 signalling pathway in human polycystic ovary syndrome.^12^ miR-202-5p promoted granulosa cell apoptosis by targeting *TGFβR2* in goat ovary.^13^ miR-320 and miR-383 directly target *E2F1/SF1* and ultimately affect the expression of their downstream gene *CYP19A1* in mouse granulosa cells.^14^

On any given day of the normal course of oestrous cycles in goats, there are 5–10 follicles ≥3 mm in diameter in the ovaries and follicles ovulate at between 6 and 9 mm in diameter.^3^^,^^15^ DF may eventually develop into mature follicles until ovulation, while SFs undergo atresia affected by various regulatory factors, among which the apoptosis of granulosa cells is the key factor leading to follicle atresia.^16^ However, the specific regulatory mechanism of miRNAs to promote follicular dominance or atresia remains unclear. In this study, we performed small RNA sequencing on DF and SF granulosa cells of goats at the first follicular wave to identify potential differentially expressed miRNAs associated with follicular dominance or atresia of a follicular wave. Our findings will provide new insights into the molecular mechanisms of regulating follicular development in goats.

## Materials and methods

### Animals and sample collection

All related experiments involving goats were conducted in strict compliance with relevant guidelines set by the Ethics Committee of Tongren University, China (Approval ID: TRXY2019-052).

Guizhou white goats were raised at Huazhen Animal Husbandry Co., Ltd. under the same conditions. Oestrus was synchronized in 15 nonlactating goats (1-year-old, 22.61 ± 0.45 kg) with two injections of prostaglandin F_2_α (PGF2α; Prostamate; IVX Animal Health) administered 14 days apart, and follicular growth was detected and recorded by daily ultrasonography, until the DF reached between 5 and 6 mm in diameter at the first follicular wave. The goats were randomly divided into three groups (*n* = 3), and ovaries were removed after slaughtering at the stages of the first follicular wave (approximately Day 3 after oestrus). DF (first scan where the growth of the largest follicle to 5–6 mm was detected by ovarian ultrasonography) and SF (first scan where a new follicle at least 3–4 mm is detected by ovarian ultrasonography) were uniformly isolated from the ovaries of each group. Granulosa cells were isolated from each group of the two types of follicles (single DF or SF granulosa cell sample containing five pooled groups) and were immediately stored at −80 °C.

### RNA isolation and library construction

Total RNA was isolated from the granulosa cells in the DF and SF of goats using TRIzol reagent (Invitrogen, CA, USA). The extracted RNAs were visualized on 1% agarose gels and were checked using the NanoPhotometer^®^ spectrophotometer (IMPLEN, CA, USA). Library construction was performed under the guidance of a Small RNA Sample Pre Kit (Illumina, CA, USA). The miRNA libraries were sequenced on an Illumina HiSeq4000 platform (Illumina, CA, USA) and single-end reads were generated.

### Data processing

Raw reads in fastq format were processed through in-house perl scripts and were cleaned with FastQC (http://www.bioinformatics.babraham.ac.uk/projects/fastqc/) by removing reads containing adapter or poly-N and low-quality reads to obtain high-quality clean reads. Unique reads were obtained by filtering to a minimum length of 18 nt and a maximum length of 35 nt of the clean reads of each sample. The unique reads were aligned to the reference genome (*Capra hircus* ARS1.97) using bowtie software. Then, the mapped reads were searched in the miRBase (v21.0) database and at most one mismatch was allowed, which were considered as known miRNAs. Ribosomal RNA (rRNA), transfer RNA (tRNA), small nuclear RNA (snRNA), small nucleolar RNA (snoRNA) and other ncRNA/repeats were discarded from the small RNA sequences. Novel miRNAs were predicated using miREvo and miRDeep2 based on the structure and energy of precursor sequences.

### Differential expression analysis

Transcript per kilobase per million was used as an indicator to calculate miRNA expressions. Differentially expressed miRNAs between DF and SF granulosa cells were selected using the threshold |log2(fold change)| ≥ 1 and a statistical significance of corrected *P* < 0.05. The expression-based sample clustering and principal component analysis were performed using DESeq2 R package (1.10.1).

### Target gene prediction and functional enrichment analysis

The target genes of differentially expressed miRNAs were predicted by miRanda, PITA and RNAhybrid, respectively. Based on the candidate target genes, Gene Ontology (GO) and Kyoto Encyclopedia of Genes and Genomes (KEGG) enrichment analysis was implemented by the cluster Profiler R package between DF and SF granulosa cells. GO terms and KEGG pathways with corrected *P* < 0.05 were defined as significantly enriched. To further explore the interactions between the differentially expressed miRNAs and target genes in follicular development, based on the functional enrichment results, the most related miRNAs and their targeted genes were screened. The regulatory networks were constructed and visualized through an open software platform known as Cytoscape3.7.1 (https://cytoscape.org/, USA).

### Quantitative real-time PCR (qPCR) for miRNA quantitation

Total RNA (1 μg) isolated from granulosa cells of DF and SF from individual goats was used for qPCR through TransScript^®^ Green miRNA Two-Step qRT-PCR SuperMix (Transgen, Beijing, China). qPCR was performed using a 20 μL reaction volume containing 10 μL of 2× *Perfectstart*^™^ Green qPCR SuperMix, 0.8 μL of forward and universal primers, 4 μL of cDNA and 5.2 μL RNase and DNase-free water. The qPCR reaction was conducted in three replicates (*n* = 3) using a LightCycler 480 platform (Roche, Basel, Switzerland) set at 94 °C for 30 s, followed by 40 cycles of 94 °C for 5 s, 60 °C for 15 s and 72 °C for 10 s. *U6* (F: CGCTTCGGCAGCACATATAC, R:TTCACGAATTTGCGTGTCAT) was used as the reference gene and the relative expression of the miRNAs was calculated using the 2^−△△CT^ method. A significant difference was statistically evaluated with Student’s *t*-test using SPSS 26.0. Primer information is listed in Table 1.

**Table 1.** The specific primers used for qPCR.

miRNAs	Primer sequences (5′ → 3′)	miRNA sequences
chi-miR-107-3p	F: ACACTCCAGCTGGGAGCAGCATTGTACAG	AGCAGCAUUGUACAGGGCUAU
chi-miR-125a-3p	F: ACACTCCAGCTGGGACAGGTGAGGTTCTT	ACAGGUGAGGUUCUUGGGAGC
chi-miR-342-5p	F: ACACTCCAGCTGGGAGGGGTGCTATCTGTGG	AGGGGUGCUAUCUGUGGUUGAGG
chi-miR-129-3p	F: ACACTCCAGCTGGGAAGCCCTTACCCCAAA	AAGCCCUUACCCCAAAAAGCAU
chi-miR-331-3p	F: ACACTCCAGCTGGGCCCCTGGGCCTATCC	CCCCUGGGCCUAUCCUAGAAC
chi-miR-1343	F: ACACTCCAGCTGGGCTCCTGGGGCCCGCAC	CUCCUGGGGCCCGCACUCUCGC
chi-miR-874-3p	F: ACACTCCAGCTGGGCTGCCCTGGCCCGAGGG	CUGCCCUGGCCCGAGGGACCGAC
chi-miR-93-3p	F: ACACTCCAGCTGGGACTGCTGAGCCAGCACT	ACUGCUGAGCCAGCACUUCCCGA
novel_296	F: ACACTCCAGCTGGGTGCCAAGCCCACGTT	UGCCAAGCCCACGUUCAAAGG

## Results

### Quality analysis of small RNA sequencing

In total, 93,304,489 raw reads were obtained from DF and SF granulosa cell samples. After filtering the low-quality sequences, a total of 92,019,573 clean reads were obtained. The ratio of clean reads to raw reads exceeded 97%, and the Q20 and Q30 values of each sample were higher than 98% (Table 2). These results indicated that the obtained data were of high quality.

**Table 2.** Quality control of small RNA sequencing data.

Sample	Raw reads	Clean reads	Clean reads/raw reads	Error rate	Q20	Q30	GC content
DF1	12,622,676	12,504,252	99.06%	0.01%	99.70%	99.10%	49.14%
DF2	15,556,673	15,216,187	97.81%	0.01%	99.67%	99.00%	49.62%
DF3	18,825,482	18,571,779	98.65%	0.01%	99.63%	98.90%	49.15%
SF1	14,227,266	13,980,174	98.26%	0.01%	99.60%	98.82%	49.33%
SF2	15,594,356	15,383,912	98.65%	0.01%	99.63%	98.87%	49.27%
SF3	16,478,036	16,363,269	99.30%	0.01%	99.65%	98.95%	49.20%

### Small RNA length distribution

**Figure 1.**
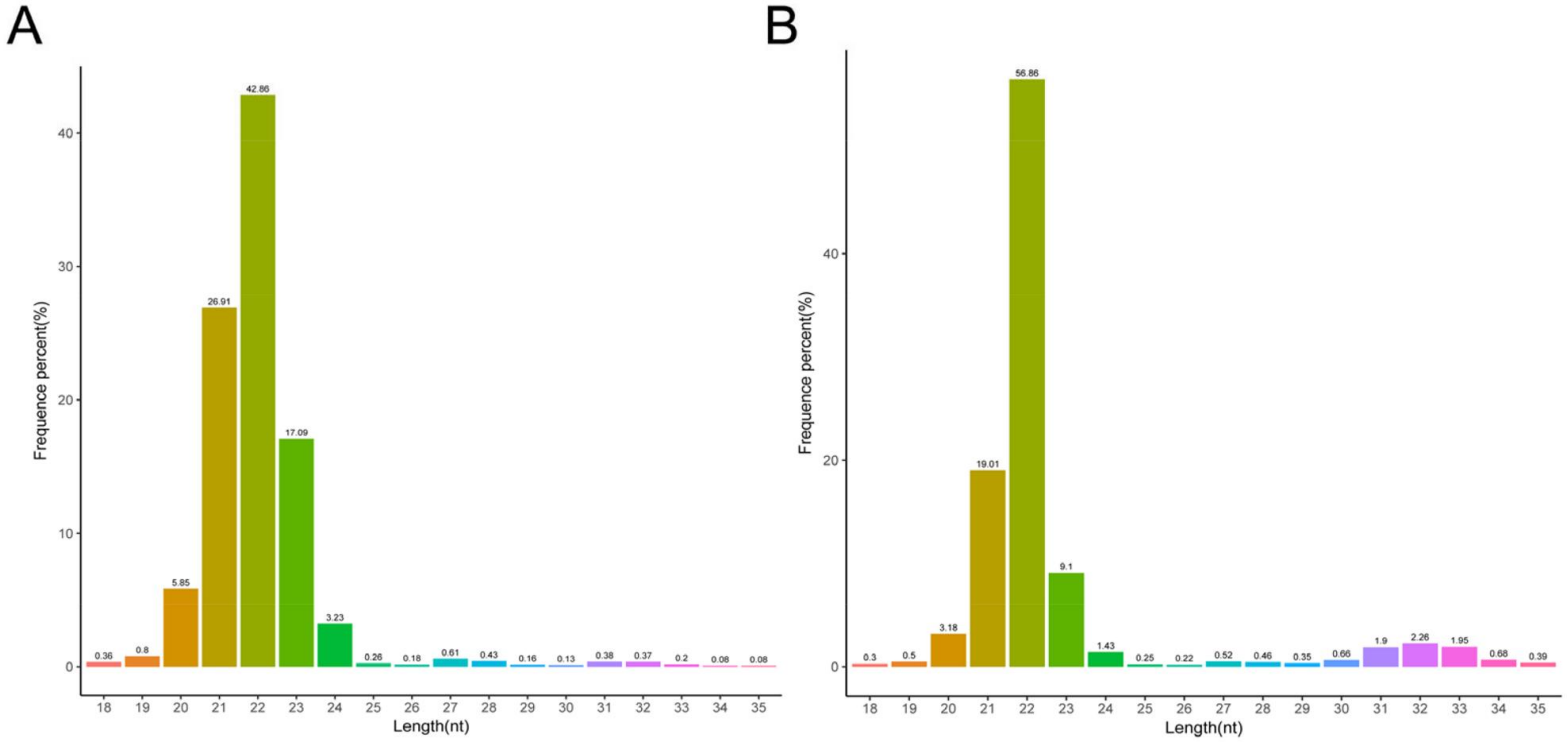
Length distribution of small RNAs in DF (A) and SF (B) granulosa cells.

Length distribution ranged from 18 to 35 nt of the clean reads in DF and SF granulosa cells were screened for length distribution statistics. As shown in Fig. 1, the length of the clean reads mainly ranged between 21 and 23 nt and the peak was at 22 nt, which was consistent with the reported length distribution of miRNA and could be used for subsequent analysis.

### Annotation classification of small RNAs

**Figure 2.**
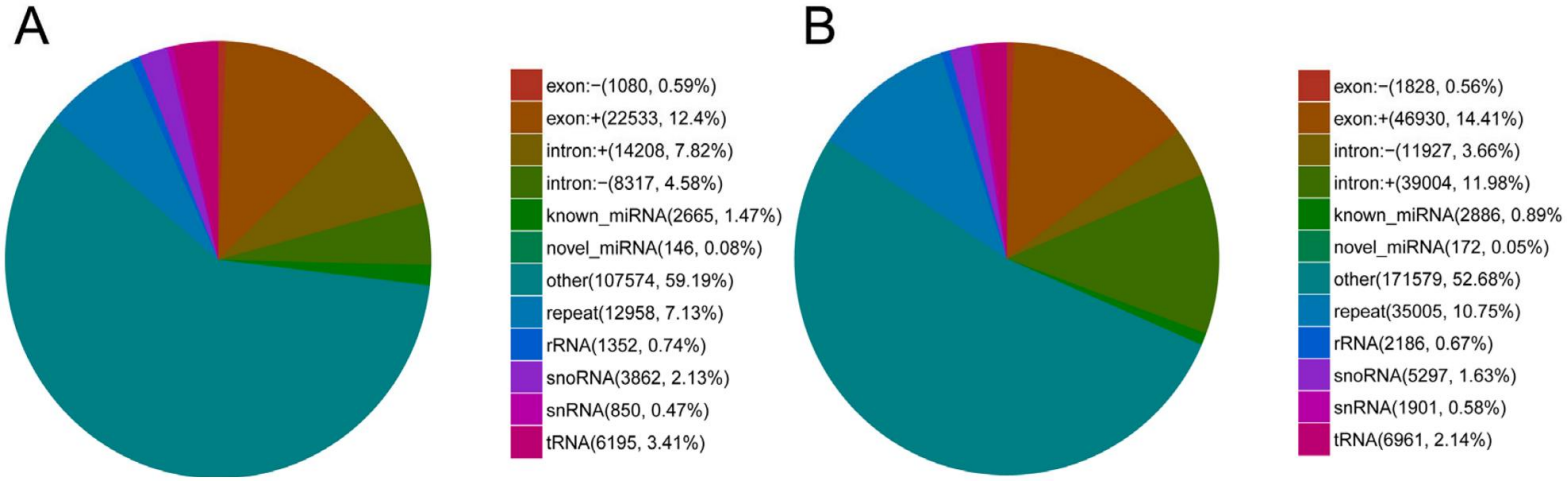
Annotation classification of small RNAs in DF (A) and SF (B) granulosa cells.

Small RNAs were annotated and classified by aligning clean reads with different databases. To make every unique small RNA mapped to only one annotation, we summarized all alignments and annotations following the priority rule: known miRNA > rRNA > tRNA  >  snRNA  >  snoRNA  >  repeat  >  gene  >  novel miRNA. As shown in Fig. 2, 2665 (1.47% of total reads) and 2886 (0.89% of total reads) unique sequences were identified as potentially conserved miRNA in DF and SF granulosa cells, respectively. One hundred and forty-six (0.08% of total reads) and 172 (0.05% of total reads) unique sequences were identified as potentially novel miRNA in DF and SF granulosa cells, respectively.

### Identification of miRNAs

In total, 402 mature miRNAs were identified from DF and SF granulosa cell samples by mapping the sequences to the reference genome with the sequences at the specified range in miRBase, including 354 known miRNAs and 48 novel miRNAs (Table S1).

### Differential expression patterns of miRNAs

**Figure 3.**
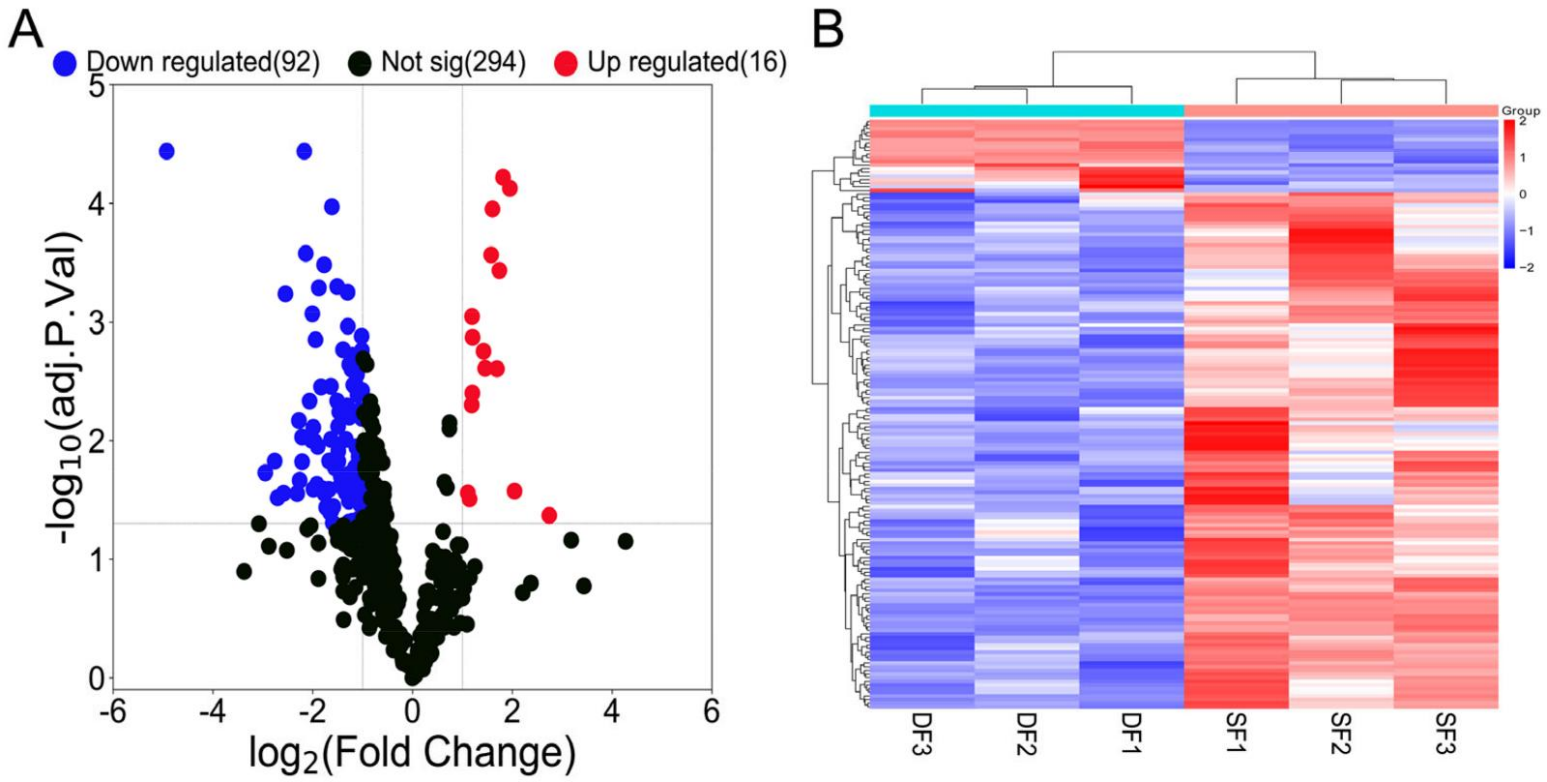
Volcano map and hierarchical clustering analysis showing the expression profiles of miRNAs in DF and SF granulosa cells. (A) Volcano map shows the miRNAs expression with corrected *P* < 0.05 and |log2(fold change)| ≥ 1. Red dots represent the upregulated miRNAs, while blue dots represent the downregulated miRNAs. (B) The heatmap shows the significantly expressed miRNAs with corrected *P* < 0.05 and |log2(fold change)| ≥ 1.

A total of 108 differentially expressed miRNAs were detected, including 16 upregulated and 92 downregulated miRNAs (Fig. 3A and Table S2). Hierarchical clustering showed distinguishable expression patterns of differentially expressed miRNAs in the samples (Fig. 3B).

### Functional enrichment analysis of differentially expressed miRNAs target genes

**Figure 4.**
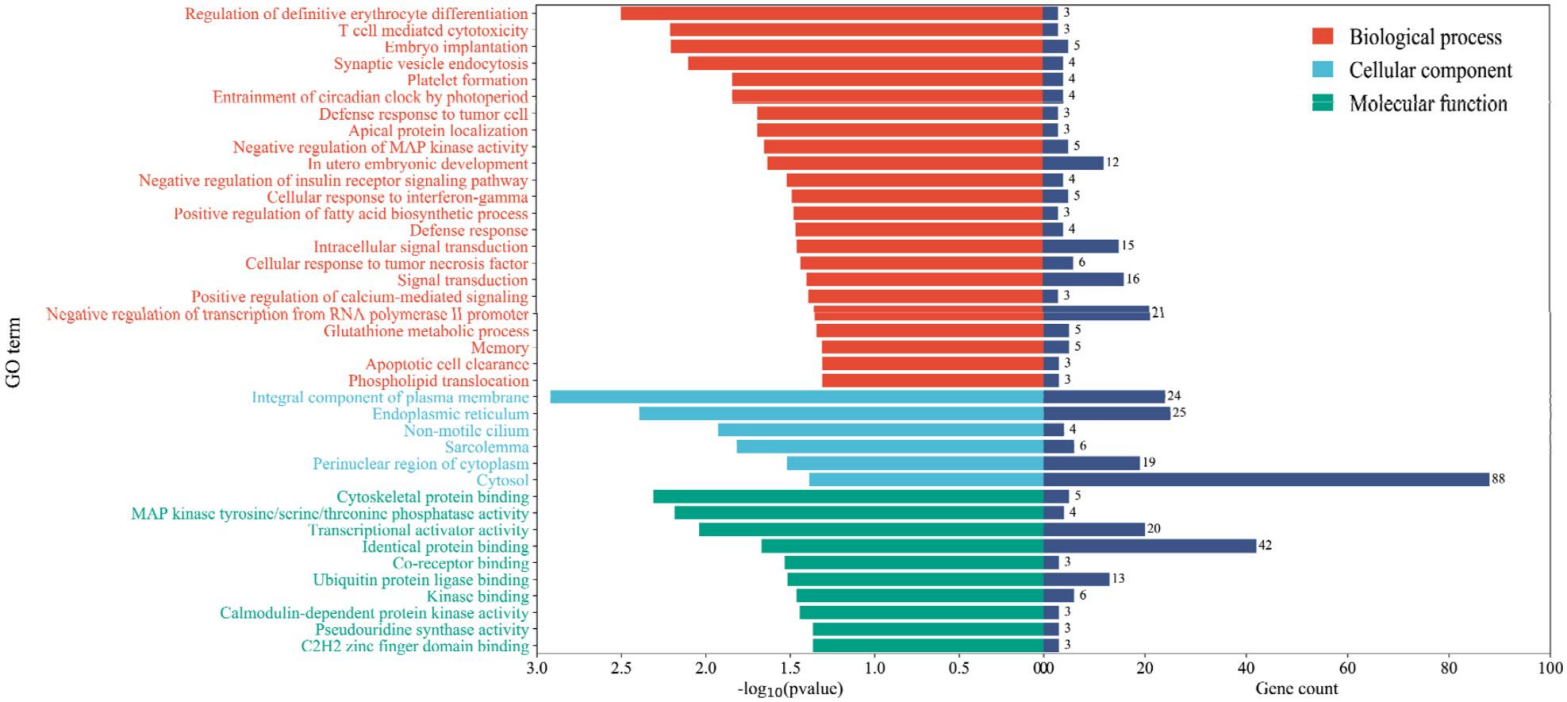
GO enrichment analysis of target genes of differentially expressed miRNAs. Gene count indicates the number of inputs of target genes of differentially expressed miRNAs annotated to the category.

**Figure 5.**
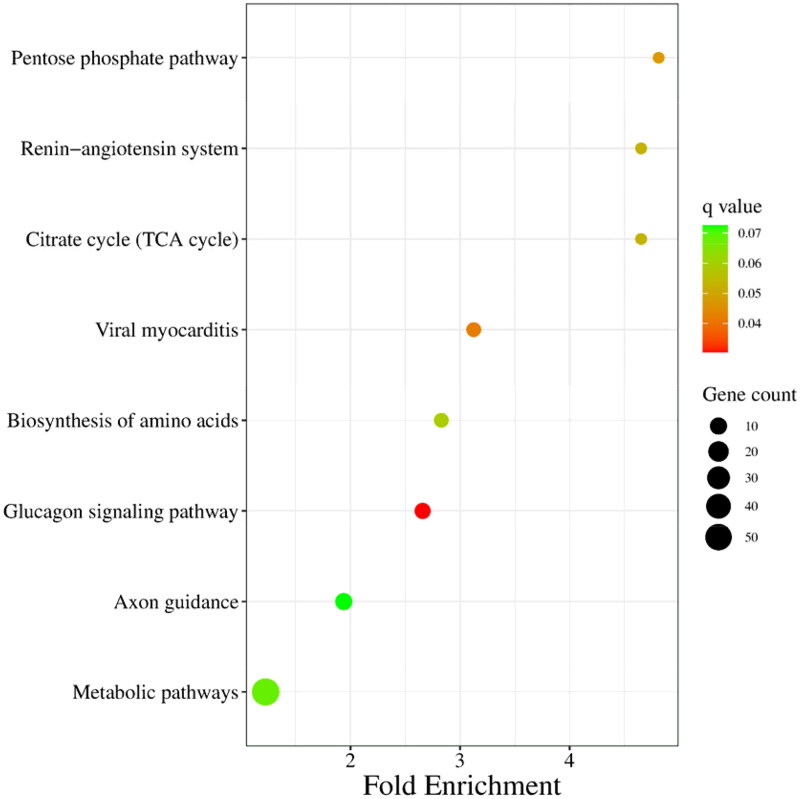
KEGG enrichment analysis of target genes of differentially expressed miRNAs. The size of each dot represents the number of target genes of differentially expressed miRNAs enriched in the corresponding pathway. A pathway with a corrected *P* value <0.05 is significantly overrepresented.

Target genes of differentially expressed miRNAs were predicted by miRanda, PITA and RNAhybrid, and 929 candidate target genes were obtained by taking intersection. GO analysis revealed that differentially expressed miRNAs target genes are involved in negative regulation of insulin receptor signalling pathway, calmodulin-dependent protein kinase activity, in utero embryo development and so forth (Fig. 4). Furthermore, a pathway enrichment analysis of differentially expressed miRNAs was conducted, and nine KEGG pathways were assigned to the target genes, which suggested that these signalling pathways were highly associated with follicular development, especially the pentose phosphate pathway (Fig. 5).

### Screening of potential functional miRNAs associated with follicular development

**Figure 6.**
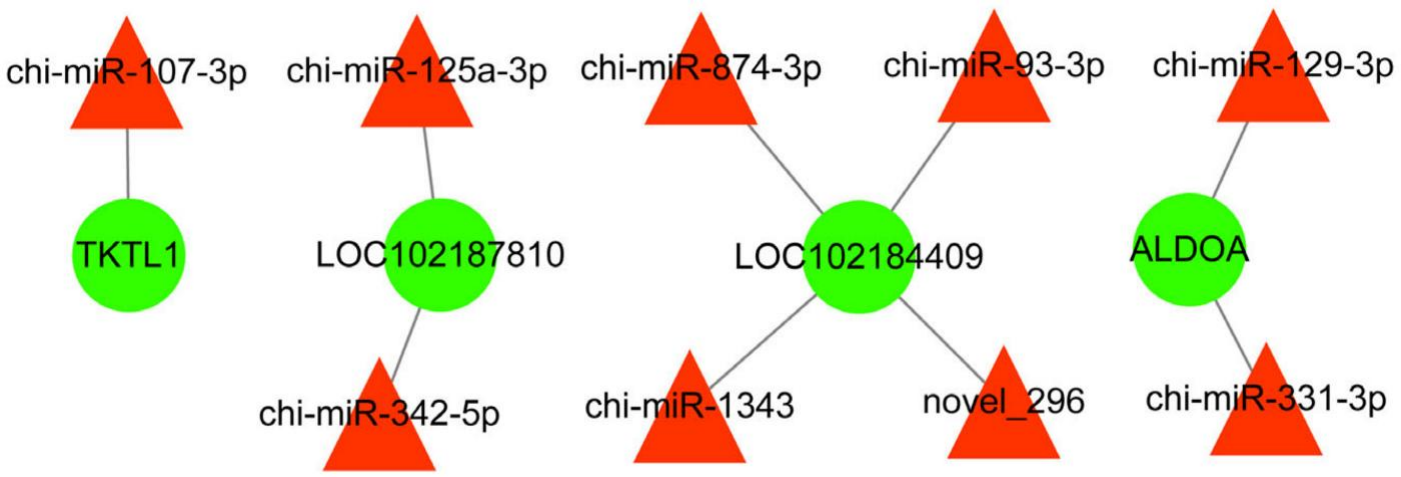
Potentially functional miRNAs and their predicted targeted genes compose this interactive network. The red triangle and green circles represent miRNAs and their targeted genes, respectively. The straight lines indicate the interaction.

To further explore the miRNAs related to follicular development and reproduction in goats, we constructed an interaction network of the miRNAs and their target genes. In total, nine differentially expressed miRNAs and four target genes were enriched in follicle development and reproduction and are shown in Fig. 6.

### Verification of differentially expressed miRNAs

**Figure 7.**
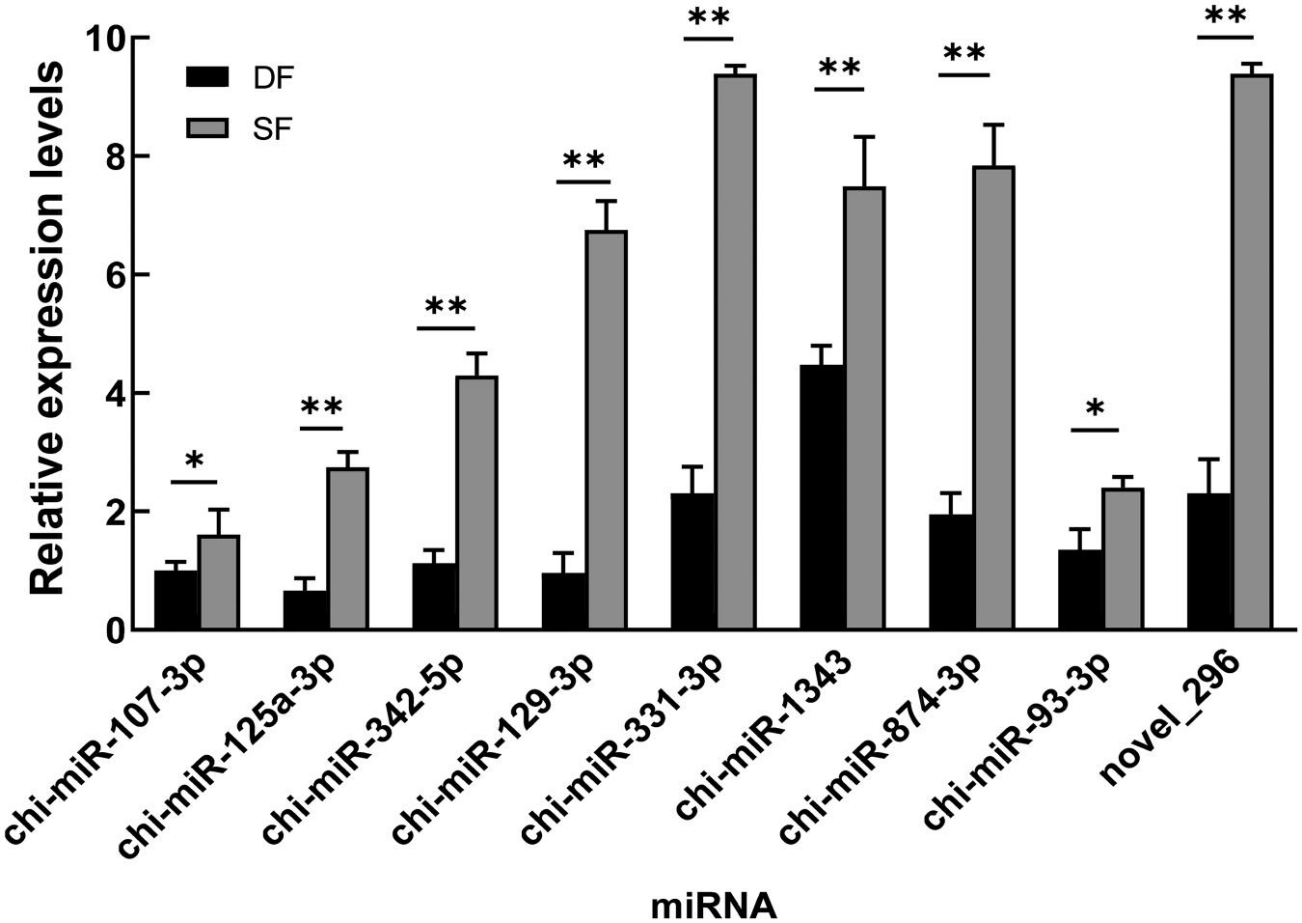
Relative expression of follicular development-related miRNAs in DF and SF granulosa cells of goats. Data is presented as the mean ± SEM (*n* = 3), * and ** indicate significant differences *P* < 0.05 and *P* < 0.01, respectively.

Nine differentially expressed miRNAs related to follicular development in Fig. 6 were selected and were validated by qPCR. The results showed that chi-miR-107-3p, chi-miR-125a-3p, chi-miR-342-5p, chi-miR-129-3p, chi-miR-331-3p, chi-miR-1343, chi-miR-874-3p, chi-miR-93-3p and novel_296 were significantly downregulated in DF granulosa cells (*P* < 0.05 or *P* < 0.01), which were consistent with the small RNA sequencing data (Fig. 7) and further indicate that the small RNA sequencing results are reliable and can represent the virtual expression pattern between DF and SF granulosa cells of Guizhou white goats.

## Discussion

Goats are born with about 1,000,000 primordial follicles in their ovaries; however, most of the follicles will be atresia at different developmental stages.^17^ Previous study has shown that the basic physical mechanism of follicular atresia is apoptosis of granulosa cells, which may result from cellular or exogenous miRNAs acting as mediators of these processes by participating in post-transcriptional regulation of mRNAs.^18^ Based on previously conducted high-throughput studies, many differentially expressed miRNAs have been shown to play important roles during follicular atresia.^19^^,^^20^ Among the most notable miRNAs that changed during atresia, miR-1275, which was shown to affect E_2_ synthesis and lead to GC apoptosis by targeting liver receptor homologue (LRH)-1.^10^,^21^ However, the molecular mechanisms governing the wave-like pattern of follicular development are incompletely described in goats.

In the present study, small RNA sequencing was performed on DF and SF granulosa cells of Guizhou white goats. Most of the sequence lengths were distributed between 21 and 23 nt, and rRNA accounted for 0.74% and 0.67%, respectively,^9^ indicating that the sequences were dominated by miRNA and the data confidence was reliable. Besides, we identified 354 known mature miRNAs and 48 novel mature miRNAs, which confirmed that miRNAs were abundant in follicular granulosa cells of goats, suggesting that the miRNAs may play crucial roles in cell proliferation, differentiation, apoptosis and degeneration. The findings provide valuable information for future studies on the regulation of miRNAs in follicular development of goats.

A total of 108 differentially expressed miRNAs were discovered, of which 16 were upregulated and 92 were downregulated. Several studies based on global miRNA expression analyses have already detected differential miRNA expression in follicles at different developmental stages of goats. miR-450-5p and miR-202-5p were significantly enriched in follicles of Guanzhong Black goat, and the expression of miR-202-5p in large follicles was 15 times that in small follicles.^22^ Another study reported that miR-183 and miR-17 families have promoted the proliferation of granulosa cells in bovine ovaries.^23^ However, many studies have shown that miRNAs can also promote apoptosis of granulosa cells. In human ovaries, miR-143-3p induced apoptosis of granulosa cells by targeting BMPR1A^24^; miR-23a and miR-27a promote apoptosis of granulosa cells by targeting SMAD5.^25^^,^^26^ Of particular interest was that the expression of miR-450-5p, miR-202-5p, miR-183 and miR-17 was higher in the DF granulosa cells than in the SF granulosa cells and might therefore potentially participate in the regulation of follicular dominance in goats, while the expression of miR-23a, miR-27a and miR-143-3p was lower in the DF granulosa cells than in the SF granulosa cells and might serve a crucial part in the follicular atresia process.

Based on the GO enrichment analysis, 929 candidate target genes of the differentially expressed miRNAs in the DF and SF granulosa cells specifically enriched in negative regulation of insulin receptor signalling pathway, calmodulin-dependent protein kinase activity, in utero embryo development and so forth. The expression of IGF enhances the E_2_ secretion of follicular granulosa cells by insulin signalling pathway and accelerates the follicular selection and dominance process.^8^ In addition, miRNA-29a is involved in the regulation of androgen/androgen receptor signalling pathways, cell proliferation, extracellular matrix formation and other processes by targeting IGF-1.^27^ Lacking the expression of Ca^2+^/calmodulin-dependent kinase IV in granulosa cells suffers abnormal follicular development and impaired fertility in mice.^28^ These results also suggested that differentially expressed miRNAs might affect the follicular dominance of goats by regulating their target genes.

In previous studies, the pentose phosphate pathway provides sufficient substrates for nuclear maturation at various stages of oocytes meiosis to regulate follicular development.^29^ Glucose can significantly promote the proliferation of follicular granulosa cells and the secretion of oestrogen in sheep.^30^ Glucose metabolism in follicular granulosa cells regulated the energy supply and promoted nuclear maturation through pentose phosphate pathway.^31^ The KEGG analysis revealed that transketolase 1 (*TKTL1*), *LOC102187810*, *LOC102184409* and aldolase A (*ALDOA*) are associated with follicular dominance involved in the pentose phosphate pathway. TKTL1 is a key enzyme linked to cell proliferation in the anaerobic pentose phosphate pathway.^32^ The increased expression of TKTL1 in ovarian cancer tissue compared to those of health was confirmed in ovarian cancer.^33^ It is possible that increased expression of TKTL1 may be associated with granulosa cell proliferation and survival during follicular dominance.^34^ ALDOA is a central enzyme in the glycolysis process which catalyses F1,6BP to glyceraldehyde 3-phosphate and dihydroxyacetone phosphate.^35^ Recently, studies have shown that downregulated lncRNA ZNF674-AS1 promoted ALDOA binding to v-ATPase, activating AMPK, thereby inhibiting the proliferation of different cell types including GCs.^36^ The aberrant protein abundance of ALDOA in prepubertal pig COCs, suggests that glucose metabolism plays an important role in oocyte competence.^37^ The remaining target proteins (LOC102187810 and LOC102184409) require further studies and clarification of their applications.

In the current study, the network between miRNAs and target genes was constructed by integrating the regulatory relationships between differentially expressed miRNAs and their target genes involved in the pentose phosphate pathway, which provides candidate miRNAs related to follicle development. These results significantly enhance the understanding of goat follicle transcriptome composition and of the potential differences in miRNA expression associated with follicular development. Yet, we were unable to identify the specific functions of these large numbers of miRNAs in the goat RNA-seq data. These need to be further studied in the future.

## Conclusions

In conclusion, we identified 108 differentially expressed miRNAs potentially associated with follicle dominance, many of which were linked to the regulation of follicular development. According to the GO and KEGG databases, the target genes of differentially expressed miRNAs were annotated with multiple biological processes associated with reproduction. The miRNA-gene transcriptional regulatory network generated in this study suggested that chi-miR-107-3p, chi-miR-125a-3p, chi-miR-342-5p, chi-miR-129-3p, chi-miR-331-3p, chi-miR-1343, chi-miR-874-3p, chi-miR-93-3p and novel_296 may play a key role in the dominance of goat follicles by regulating their target genes which involved in the pentose phosphate pathway. Our findings provide new insight for exploring the molecular mechanism of miRNAs in regulating follicular development in goats.

## Supplementary Material

Supplemental Material

Supplemental Material

## Data Availability

All datasets used in this study are available from the corresponding author on reasonable request.
