# Supplementary material for: Deciphering the miRNA transcriptome of granulosa cells from dominant and subordinate follicles at first follicular wave in goat
Source: Anim Biotechnol. 2023 Sep 26;35(1):2259967. doi: 10.1080/10495398.2023.2259967 (PMC12674301; doi:10.1080/10495398.2023.2259967)
Supplement: Supplemental Material [file LABT_A_2259967_SM9611.docx]

Table S2RNA-seq data of differentially expressed miRNAs in DF vs.SF granulosa cells

| Gene name | Log2FC | adj.P.Val |
| --- | --- | --- |
| chi-miR-429 | 2.740084575 | 0.042790536 |
| novel_456 | 2.04405164 | 0.026617507 |
| chi-miR-133b | 1.950781319 | 7.46E-05 |
| chi-miR-129-5p | 1.813635549 | 6.03E-05 |
| chi-miR-135b-5p | 1.740492849 | 0.000367414 |
| novel_399 | 1.691963521 | 0.002484873 |
| chi-miR-135a | 1.601875769 | 0.000111622 |
| chi-miR-1468-5p | 1.575481702 | 0.000271889 |
| chi-miR-133a-3p | 1.454297629 | 0.002462266 |
| novel_378 | 1.422975638 | 0.001767719 |
| chi-miR-181d | 1.202485715 | 0.001348314 |
| chi-miR-1306-3p | 1.198050698 | 0.00398108 |
| chi-miR-145-3p | 1.192430215 | 0.000896628 |
| chi-miR-181c-5p | 1.18500262 | 0.004992118 |
| novel_185 | 1.142375179 | 0.03102652 |
| chi-miR-29b-5p | 1.108149965 | 0.027589001 |
| chi-let-7d-3p | -1.00988801 | 0.003788011 |
| chi-miR-140-3p | -1.0147515 | 0.001738833 |
| chi-miR-1343 | -1.01655531 | 0.006467559 |
| chi-miR-106b-3p | -1.02083512 | 0.001318813 |
| chi-miR-491-5p | -1.03438457 | 0.015008264 |
| chi-miR-485-5p | -1.03904868 | 0.048524827 |
| chi-miR-2483-5p | -1.04140145 | 0.034709523 |
| chi-miR-10a-3p | -1.04384268 | 0.019631009 |
| chi-miR-193b-3p | -1.05062226 | 0.027332957 |
| chi-let-7e-3p | -1.05938941 | 0.005556896 |
| chi-miR-154b-5p | -1.06173697 | 0.013255949 |
| chi-miR-15b-5p | -1.07290664 | 0.003972329 |
| novel_428 | -1.0781392 | 0.045767873 |
| chi-miR-133a-5p | -1.08000159 | 0.013607399 |
| chi-miR-193a | -1.0806882 | 0.023592568 |
| chi-miR-19b-5p | -1.08319947 | 0.016017469 |
| chi-miR-19a | -1.09121929 | 0.015565211 |
| chi-miR-34b-3p | -1.09285958 | 0.011917221 |
| chi-miR-128-3p | -1.1026067 | 0.004098017 |
| chi-miR-379-3p | -1.11217304 | 0.002762582 |
| chi-let-7c-3p | -1.12542089 | 0.020146579 |
| chi-miR-134 | -1.12952779 | 0.011266613 |
| chi-miR-495-3p | -1.13556649 | 0.049016125 |
| chi-miR-1 | -1.14832928 | 0.003260871 |
| chi-miR-125a-3p | -1.15339988 | 0.016918122 |
| novel_272 | -1.15419887 | 0.022788339 |
| chi-miR-331-3p | -1.1855372 | 0.049966971 |
| chi-miR-136-3p | -1.18785652 | 0.003398104 |
| chi-miR-424-5p | -1.20326853 | 0.001915184 |
| chi-miR-411b-5p | -1.20465282 | 0.047766233 |
| chi-miR-127-3p | -1.22286182 | 0.002508545 |
| chi-miR-193b-5p | -1.26764806 | 0.002284259 |
| novel_296 | -1.26782852 | 0.019053782 |
| novel_333 | -1.26873529 | 0.006319551 |
| chi-miR-502b-5p | -1.27351969 | 0.04858848 |
| chi-miR-33a-3p | -1.27638003 | 0.032360675 |
| chi-miR-127-5p | -1.29672254 | 0.001085742 |
| chi-miR-1197-3p | -1.30279952 | 0.000561693 |
| chi-miR-126-3p | -1.30897931 | 0.005089852 |
| chi-miR-184 | -1.33551322 | 0.027456671 |
| chi-miR-874-3p | -1.33664548 | 0.009799189 |
| chi-miR-381 | -1.33720457 | 0.021145612 |
| chi-let-7i-3p | -1.39233476 | 0.00171592 |
| chi-miR-105b-5p | -1.43935291 | 0.01990573 |
| chi-miR-369-5p | -1.45047245 | 0.023921483 |
| chi-miR-329a-3p | -1.46852178 | 0.005732949 |
| chi-miR-16a-3p | -1.48693139 | 0.014970602 |
| chi-miR-338-5p | -1.48804142 | 0.012257225 |
| chi-miR-101-5p | -1.49155363 | 0.007590599 |
| chi-miR-379-5p | -1.50972663 | 0.000501777 |
| chi-miR-92a-5p | -1.51099532 | 0.004631515 |
| chi-miR-34c-5p | -1.55486958 | 0.016967297 |
| chi-miR-21-3p | -1.56552345 | 0.015047065 |
| chi-miR-301a-3p | -1.5872218 | 0.035966426 |
| chi-miR-542-3p | -1.59798223 | 0.049356521 |
| chi-miR-93-3p | -1.61629476 | 0.000106902 |
| chi-miR-383 | -1.62577813 | 0.038903669 |
| novel_261 | -1.63476709 | 0.003477147 |
| chi-miR-15b-3p | -1.63536077 | 0.009791462 |
| chi-miR-582-5p | -1.65120836 | 0.040514264 |
| chi-miR-34c-3p | -1.66621996 | 0.036555812 |
| chi-miR-16b-3p | -1.67153748 | 0.014827654 |
| chi-miR-1814 | -1.67657906 | 0.02572101 |
| chi-miR-433 | -1.72993096 | 0.036528111 |
| chi-miR-655 | -1.76194786 | 0.027975346 |
| chi-miR-105b-3p | -1.7720667 | 0.000329906 |
| chi-miR-146b-3p | -1.79881253 | 0.025477837 |
| novel_233 | -1.83372963 | 0.003518218 |
| novel_215 | -1.8390894 | 0.025581627 |
| chi-miR-17-3p | -1.87782426 | 0.000514647 |
| chi-miR-483 | -1.89885679 | 0.011151105 |
| chi-miR-432-5p | -1.91717955 | 0.023497316 |
| chi-miR-130b-5p | -1.944117 | 0.00141305 |
| chi-miR-656 | -1.95380227 | 0.010296481 |
| chi-miR-324-5p | -1.98700895 | 0.025607645 |
| chi-miR-301a-5p | -1.98865989 | 0.007762344 |
| chi-miR-410-3p | -2.0080072 | 0.000850554 |
| chi-miR-301b | -2.02103573 | 0.009941216 |
| chi-miR-409-5p | -2.06338044 | 0.0046321 |
| chi-miR-153 | -2.1391542 | 0.00026449 |
| novel_98 | -2.16926504 | 3.64E-05 |
| chi-miR-493-5p | -2.21292352 | 0.015052376 |
| chi-miR-154a-3p | -2.21304757 | 0.009344062 |
| chi-miR-323b | -2.26246875 | 0.021687894 |
| chi-miR-106a-5p | -2.27629704 | 0.006772665 |
| chi-miR-450-3p | -2.31208279 | 0.027933432 |
| chi-miR-100-3p | -2.54712403 | 0.000579692 |
| chi-miR-542-5p | -2.58367556 | 0.027783545 |
| chi-miR-141 | -2.70440192 | 0.03037288 |
| chi-miR-130b-3p | -2.76226928 | 0.014857615 |
| chi-miR-412-5p | -2.95104686 | 0.018645968 |
| novel_308 | -4.9266204 | 3.64E-05 |
