# Supplementary material for: Deciphering the miRNA transcriptome of granulosa cells from dominant and subordinate follicles at first follicular wave in goat
Source: Anim Biotechnol. 2023 Sep 26;35(1):2259967. doi: 10.1080/10495398.2023.2259967 (PMC12674301; doi:10.1080/10495398.2023.2259967)
Supplement: Supplemental Material [file LABT_A_2259967_SM9610.docx]

| **Table S1 miRNA identified in DF and SF granulosa cells of goat** | | | | | | |
| --- | --- | --- | --- | --- | --- | --- |
| **miRNAs** | **DF1** | **DF2** | **DF3** | **SF1** | **SF2** | **SF3** |
| chi-let-7a-3p | 10.38362367 | 9.515459 | 9.340727 | 14.44642 | 12.02512 | 15.71195 |
| chi-let-7a-5p | 3500.600312 | 3777.049 | 3091.693 | 6204.739 | 6213.098 | 5025.624 |
| chi-let-7b-3p | 5.399484308 | 5.631351 | 6.198053 | 7.175535 | 7.652346 | 8.484452 |
| chi-let-7b-5p | 9599.452409 | 5811.057 | 7099.637 | 4767.456 | 5498.757 | 4226.304 |
| chi-let-7c-3p | 1.522931471 | 1.164829 | 2.182413 | 2.862027 | 2.793714 | 5.027823 |
| chi-let-7c-5p | 7708.94066 | 9917.71 | 8548.498 | 5944.022 | 4941.463 | 5902.455 |
| chi-let-7d-3p | 8.045003456 | 9.926054 | 8.816948 | 21.39706 | 15.54762 | 17.28314 |
| chi-let-7d-5p | 60.97507487 | 80.56519 | 76.99553 | 121.0229 | 158.6344 | 107.4697 |
| chi-let-7e-3p | 4.153449467 | 5.06991 | 6.285349 | 9.540091 | 12.75391 | 9.846154 |
| chi-let-7e-5p | 591.9015488 | 653.5371 | 504.7939 | 821.6744 | 778.9603 | 841.8462 |
| chi-let-7f-3p | 1.099828103 | 1.294703 | 0.960262 | 1.771731 | 1.579056 | 1.547463 |
| chi-let-7f-5p | 25738.511 | 14203.61 | 17219.77 | 14571.94 | 13045.31 | 12226.83 |
| chi-let-7g-3p | 0.415344947 | 0.294703 | 0.611076 | 2.453166 | 1.214658 | 1.256956 |
| chi-let-7g-5p | 17407.38361 | 17230.91 | 17276.6 | 10594 | 11309.8 | 9306.396 |
| chi-let-7i-3p | 0.969138209 | 1.596396 | 1.396744 | 3.816037 | 3.259675 | 3.14239 |
| chi-let-7i-5p | 28721.37996 | 27315.64 | 27334.89 | 19469.15 | 13294.55 | 16639.27 |
| chi-miR-1 | 1614.811701 | 1770.951 | 1585.916 | 3126.152 | 3276.12 | 4822.625 |
| chi-miR-100-3p | 0.138448316 | 0.287712 | 0.349186 | 1.226583 | 1.485863 | 1.523732 |
| chi-miR-100-5p | 6213.00661 | 6023.964 | 6053.577 | 5034.034 | 5986.321 | 5060.308 |
| chi-miR-101-3p | 6924.492504 | 5674.969 | 6254.239 | 5506.95 | 6636.528 | 6077.905 |
| chi-miR-101-5p | 0.692241578 | 1.596396 | 1.571337 | 3.36287 | 3.036645 | 3.780687 |
| chi-miR-103-3p | 1863.791224 | 709.2093 | 978.996 | 957.1437 | 580.4851 | 539.2864 |
| chi-miR-105a | 4.984139361 | 4.17182 | 4.658634 | 3.812665 | 3.158111 | 3.456628 |
| chi-miR-105b-3p | 0.138448316 | 0.143856 | 0.136483 | 0.408861 | 0.421466 | 0.628478 |
| chi-miR-105b-5p | 0.276896631 | 0.143856 | 0.349186 | 0.545148 | 0.485863 | 1.047463 |
| chi-miR-106a-5p | 0.138448316 | 0.463135 | 0.523779 | 1.226583 | 1.485863 | 2.094926 |
| chi-miR-106b-3p | 42.48347481 | 43.79805 | 44.78311 | 87.76884 | 84.98421 | 93.32897 |
| chi-miR-106b-5p | 90.12985344 | 97.82198 | 72.80529 | 112.3005 | 160.0919 | 117.5254 |
| chi-miR-107-3p | 51.91811834 | 70.63322 | 46.61634 | 115.0262 | 111.2627 | 101.7087 |
| chi-miR-10a-3p | 2.450894044 | 2.589405 | 3.142675 | 8.722369 | 4.251304 | 4.713584 |
| chi-miR-10a-5p | 811.9251626 | 799.407 | 1140.878 | 730.1613 | 612.8025 | 816.5303 |
| chi-miR-10b-3p | 17.30603945 | 24.23769 | 24.35573 | 36.93378 | 32.30991 | 33.72831 |
| chi-miR-10b-5p | 7653.976679 | 22683.19 | 12649.18 | 18114.73 | 32139.57 | 16530.64 |
| chi-miR-1197-3p | 0.719279259 | 0.719279 | 0.785669 | 1.908018 | 1.607329 | 1.99018 |
| chi-miR-122 | 0.138448316 | 0.575423 | 0.360262 | 0.545148 | 0.971727 | 0.620295 |
| chi-miR-1248-5p | 0.692241578 | 0.863135 | 0.66189 | 1.36287 | 0.971727 | 1.209493 |
| chi-miR-125a-3p | 1.676380939 | 1.72627 | 2.531599 | 4.088611 | 3.036645 | 6.494272 |
| chi-miR-125a-5p | 728.6534849 | 653.9915 | 656.5571 | 1257.793 | 1305.758 | 965.1326 |
| chi-miR-125b-3p | 120.0346896 | 107.4401 | 128.3259 | 226.3727 | 228.9631 | 249.1915 |
| chi-miR-125b-5p | 3193.033502 | 3231.346 | 4090.191 | 5819.592 | 7467.84 | 6147.771 |
| chi-miR-126-3p | 5298.538872 | 7458.638 | 5192.222 | 18853.95 | 15789.79 | 10483.74 |
| chi-miR-126-5p | 88.60692197 | 70.63322 | 50.0209 | 138.3313 | 90.49203 | 93.64321 |
| chi-miR-1271-3p | 0.261889545 | 0.287712 | 0.26189 | 0.408861 | 0.485863 | 0.404746 |
| chi-miR-1271-5p | 91.79123323 | 82.4552 | 83.80465 | 165.9976 | 160.8207 | 161.3093 |
| chi-miR-127-3p | 364.3381506 | 315.4759 | 502.3914 | 959.5969 | 923.6728 | 828.4386 |
| chi-miR-127-5p | 12.39948431 | 13.09088 | 12.83259 | 38.29665 | 30.92039 | 26.08183 |
| chi-miR-128-3p | 76.28502189 | 56.37938 | 47.31471 | 135.0604 | 123.2208 | 121.0867 |
| chi-miR-129-3p | 1.107586525 | 0.863135 | 0.611076 | 1.36287 | 1.728795 | 1.780687 |
| chi-miR-129-5p | 27.13586985 | 26.61737 | 25.15049 | 7.086925 | 7.494235 | 7.873977 |
| chi-miR-1296 | 2.353621365 | 1.582414 | 1.698372 | 3.407175 | 2.793714 | 2.571195 |
| chi-miR-1306-3p | 1.661379787 | 1.431568 | 0.960262 | 0.545148 | 0.607329 | 0.571195 |
| chi-miR-1306-5p | 2.630517996 | 2.157838 | 1.571337 | 3.226583 | 3.91518 | 3.247136 |
| chi-miR-1307-3p | 16.8906945 | 24.02393 | 19.60262 | 43.47556 | 35.02196 | 32.6252 |
| chi-miR-1307-5p | 2.291897783 | 2.877117 | 2.182413 | 4.497472 | 4.251304 | 4.085106 |
| chi-miR-130a-3p | 45.07070832 | 46.74508 | 43.47366 | 68.55237 | 68.22604 | 67.98036 |
| chi-miR-130a-5p | 1.630517996 | 1.020973 | 1.484041 | 2.862027 | 2.186385 | 2.618658 |
| chi-miR-130b-3p | 0.553793262 | 0.870126 | 0.095116 | 2.316879 | 2.45759 | 2.513912 |
| chi-miR-130b-5p | 2.076724734 | 4.041946 | 4.714012 | 12.67469 | 14.12984 | 12.5892 |
| chi-miR-133a-3p | 190.9202272 | 125.8941 | 121.6495 | 42.11269 | 50.89212 | 66.30442 |
| chi-miR-133a-5p | 0.891553988 | 1.438559 | 0.785669 | 2.545148 | 1.700521 | 2.199673 |
| chi-miR-133b | 6.783967464 | 6.575423 | 6.785669 | 1.771731 | 1.485863 | 1.99018 |
| chi-miR-134 | 1.415344947 | 1.72627 | 2.269709 | 3.316879 | 3.214658 | 5.446809 |
| chi-miR-1343 | 2.076724734 | 2.315676 | 2.531599 | 3.543462 | 5.22303 | 5.446809 |
| chi-miR-135a | 15.49245955 | 15.10486 | 16.49186 | 5.13123 | 5.239774 | 5.13257 |
| chi-miR-135b-5p | 4.153449467 | 4.006991 | 4.523779 | 1.36287 | 0.971727 | 1.523732 |
| chi-miR-136-3p | 14.2601765 | 21.29067 | 21.16486 | 48.10932 | 36.68268 | 43.05074 |
| chi-miR-136-5p | 37.65794184 | 30.13982 | 33.43457 | 40.06838 | 46.64082 | 55.51555 |
| chi-miR-1388-3p | 6.922415779 | 7.048937 | 6.444302 | 5.996629 | 6.437688 | 6.599018 |
| chi-miR-1388-5p | 8.168450619 | 9.926054 | 8.01564 | 13.21984 | 11.17486 | 11.3126 |
| chi-miR-140-3p | 692.0521806 | 609.6839 | 664.9376 | 1253.704 | 1278.671 | 1443.718 |
| chi-miR-140-5p | 166.8302203 | 121.1064 | 177.3447 | 273.9022 | 212.5652 | 226.743 |
| chi-miR-141 | 0.07629499 | 0.575423 | 0.087297 | 0.408861 | 1.45759 | 1.780687 |
| chi-miR-143-3p | 232225.1746 | 65289.7 | 34957.37 | 68632.1 | 85419.98 | 52616.07 |
| chi-miR-143-5p | 143.4271385 | 230.4571 | 136.8809 | 254.5842 | 273.1766 | 237.7741 |
| chi-miR-144-3p | 0.415344947 | 0.450847 | 0.436483 | 0.545148 | 0.850261 | 0.809165 |
| chi-miR-144-5p | 0.830689893 | 1.438559 | 1.222151 | 2.272574 | 2.186385 | 2.199673 |
| chi-miR-145-3p | 994.1973542 | 876.6146 | 878.0394 | 429.9855 | 356.7451 | 417.9378 |
| chi-miR-145-5p | 3618.371095 | 4788.098 | 1738.074 | 5932.562 | 5680.349 | 2428.439 |
| chi-miR-1468-5p | 80.8538163 | 84.23769 | 82.4352 | 25.28148 | 25.26489 | 33.20458 |
| chi-miR-146a | 48.97038757 | 59.55632 | 45.1323 | 59.42114 | 77.61666 | 62.53355 |
| chi-miR-146b-3p | 0.522931471 | 0.143856 | 0.698372 | 1.545148 | 1.242932 | 1.152209 |
| chi-miR-146b-5p | 38.53191621 | 48.19171 | 34.30753 | 53.49289 | 63.48476 | 63.58101 |
| chi-miR-147-3p | 0.922415779 | 1.438559 | 1.484041 | 1.771731 | 1.579056 | 1.99018 |
| chi-miR-148a-3p | 575901.4924 | 583246.3 | 607410.6 | 484081.3 | 471396.2 | 513096.6 |
| chi-miR-148a-5p | 155.2408779 | 204.9946 | 129.4607 | 244.2263 | 219.8531 | 245.5254 |
| chi-miR-148b-3p | 705.6710645 | 820.2459 | 795.2713 | 1124.368 | 945.9758 | 947.9935 |
| chi-miR-148b-5p | 14.09000691 | 16.68728 | 9.77721 | 22.0785 | 18.58427 | 16.02619 |
| chi-miR-150 | 16.27345868 | 12.3716 | 14.31663 | 17.30845 | 19.07013 | 24.51064 |
| chi-miR-151-3p | 1801.574767 | 2276.087 | 1710.837 | 1209.423 | 1498.159 | 1655.646 |
| chi-miR-151-5p | 59.99140513 | 66.4614 | 54.73491 | 107.5967 | 106.2826 | 101.2766 |
| chi-miR-153 | 1.276896631 | 1.336648 | 1.484041 | 7.717312 | 4.251304 | 6.599018 |
| chi-miR-154a-3p | 0.138448316 | 0.438559 | 0.523779 | 1.226583 | 1.121466 | 2.304419 |
| chi-miR-154b-5p | 1.276896631 | 1.870126 | 1.134855 | 3.587768 | 2.186385 | 3.14239 |
| chi-miR-155-5p | 122.9999203 | 178.0935 | 127.2783 | 215.4698 | 243.3297 | 219.1293 |
| chi-miR-15a-5p | 5.522071984 | 6.329657 | 5.936163 | 8.858656 | 10.93192 | 9.436989 |
| chi-miR-15b-3p | 0.276896631 | 0.719279 | 0.447558 | 0.954009 | 1.485863 | 1.885434 |
| chi-miR-15b-5p | 15.78310798 | 12.00995 | 12.83259 | 24.66795 | 26.39789 | 34.77578 |
| chi-miR-16a-3p | 0.415344947 | 0.506991 | 0.174593 | 1.136287 | 0.850261 | 0.837971 |
| chi-miR-16a-5p | 459.562459 | 567.9429 | 418.3249 | 732.9516 | 573.4401 | 775.2275 |
| chi-miR-16b-3p | 0.24603484 | 0.719279 | 0.26189 | 1.635444 | 0.971727 | 0.942717 |
| chi-miR-16b-5p | 275.0968031 | 262.1054 | 156.6099 | 311.5521 | 349.4908 | 315.8101 |
| chi-miR-17-3p | 0.384483156 | 0.589405 | 0.611076 | 2.316879 | 1.821987 | 1.628478 |
| chi-miR-17-5p | 82.6536444 | 181.4022 | 102.8353 | 177.7183 | 154.1401 | 203.7316 |
| chi-miR-1814 | 0.692241578 | 0.143856 | 0.26189 | 0.954009 | 0.971727 | 0.918985 |
| chi-miR-181b-5p | 42.22673625 | 74.22962 | 33.60916 | 74.95786 | 76.68473 | 75.35516 |
| chi-miR-181c-3p | 4.568794414 | 3.013982 | 3.182413 | 1.090296 | 3.158111 | 2.199673 |
| chi-miR-181c-5p | 5.122587677 | 5.178811 | 5.74593 | 2.589453 | 2.98847 | 1.675941 |
| chi-miR-181d | 37.65794184 | 35.38854 | 34.75311 | 18.9439 | 14.61776 | 13.72177 |
| chi-miR-182 | 8.949839621 | 7.768216 | 4.714012 | 4.224898 | 3.519351 | 5.656301 |
| chi-miR-183 | 3.429486611 | 6.041946 | 2.531599 | 5.817722 | 6.68062 | 4.085106 |
| chi-miR-1839 | 0.415344947 | 0.287712 | 0.174593 | 0.272574 | 0.104746 | 0.104746 |
| chi-miR-184 | 0.384483156 | 0.3 | 0.26189 | 1.36287 | 0.850261 | 0.418985 |
| chi-miR-186-5p | 1196.885688 | 1126.247 | 426.6181 | 1231.284 | 1185.263 | 730.6056 |
| chi-miR-187 | 2.075865247 | 0.143856 | 0.785669 | 6.269203 | 0.485863 | 1.361702 |
| chi-miR-188-5p | 1.107586525 | 2.157838 | 2.095116 | 2.316879 | 3.036645 | 2.99018 |
| chi-miR-18a-3p | 0.27 | 0.287712 | 0.26189 | 0.136287 | 0.721466 | 0.628478 |
| chi-miR-18a-5p | 1.522931471 | 5.754234 | 4.801308 | 6.40549 | 6.802086 | 8.274959 |
| chi-miR-190a-5p | 2.630517996 | 2.589405 | 2.095116 | 3.771731 | 3.401043 | 3.666121 |
| chi-miR-190b | 4.153449467 | 4.891099 | 8.571337 | 2.044305 | 3.522509 | 7.018003 |
| chi-miR-191-3p | 2.630517996 | 1.294703 | 0.26189 | 2.944305 | 1.943453 | 0.942717 |
| chi-miR-191-5p | 1366.06953 | 1026.699 | 916.8827 | 1016.429 | 916.3381 | 899.2471 |
| chi-miR-192-5p | 372.5644172 | 344.3909 | 382.6243 | 293.426 | 307.9158 | 329.1129 |
| chi-miR-193a | 3.351902391 | 4.459531 | 4.801308 | 8.586082 | 13.48271 | 5.865794 |
| chi-miR-193b-3p | 3.351902391 | 4.747243 | 4.801308 | 8.586082 | 13.48271 | 5.865794 |
| chi-miR-193b-5p | 0.322415779 | 0.287712 | 0.3 | 0.545148 | 0.850261 | 0.837971 |
| chi-miR-194 | 144.4015932 | 155.652 | 180.2255 | 130.4267 | 152.804 | 125.6956 |
| chi-miR-195-3p | 214.8717858 | 111.4883 | 149.9336 | 140.1031 | 75.91613 | 102.1277 |
| chi-miR-195-5p | 28.65880133 | 34.81312 | 17.19741 | 37.88779 | 50.89418 | 30.48118 |
| chi-miR-196a | 0.138448316 | 4.603387 | 3.317268 | 6.40549 | 13.23977 | 9.9509 |
| chi-miR-196b | 0.415344947 | 5.178811 | 11.95962 | 13.6287 | 29.51619 | 38.54664 |
| chi-miR-197-3p | 16.47534955 | 13.37859 | 9.77721 | 27.93884 | 21.74238 | 14.97872 |
| chi-miR-199a-3p | 3949.828546 | 3893.89 | 4495.334 | 4500.879 | 4962.364 | 5437.696 |
| chi-miR-199a-5p | 3137.238831 | 3850.014 | 2798.464 | 3852.834 | 4474.801 | 3593.322 |
| chi-miR-199b-5p | 1138.460499 | 1032.166 | 865.8068 | 1821.203 | 1145.423 | 1148.229 |
| chi-miR-19a | 5.676380939 | 10.49744 | 9.340727 | 19.21647 | 20.89212 | 13.40753 |
| chi-miR-19b-3p | 61.74794875 | 165.8658 | 50.80657 | 126.6106 | 181.8343 | 94.79542 |
| chi-miR-19b-5p | 0.276896631 | 0.150847 | 0.26189 | 0.545148 | 0.364397 | 0.523732 |
| chi-miR-200a | 825.0135126 | 51.72223 | 14.57852 | 11.03925 | 33.4031 | 12.15057 |
| chi-miR-200b | 12.95459781 | 20.28368 | 17.19741 | 15.12786 | 36.31828 | 30.27169 |
| chi-miR-200c | 1.247204452 | 4.603387 | 1.396744 | 1.499157 | 11.78218 | 6.180033 |
| chi-miR-202-3p | 1.5 | 1.72627 | 1.134855 | 2.316879 | 2.186385 | 10.16039 |
| chi-miR-202-5p | 4.568794414 | 759.8466 | 333.7346 | 912.7142 | 758.4326 | 1867.627 |
| chi-miR-204-3p | 0.138448316 | 0.143856 | 0.523779 | 0.817722 | 0.242932 | 0.942717 |
| chi-miR-204-5p | 24.36690354 | 40.27964 | 31.95052 | 43.33927 | 40.69105 | 45.87889 |
| chi-miR-206 | 67.14743306 | 41.28663 | 62.17331 | 65.96292 | 4.372769 | 61.90507 |
| chi-miR-20a-3p | 0.692241578 | 1.150847 | 0.611076 | 1.36287 | 2.429316 | 1.152209 |
| chi-miR-20a-5p | 285.2035301 | 293.7133 | 256.3899 | 357.072 | 416.3848 | 497.964 |
| chi-miR-20b | 0.553793262 | 1.582414 | 1.134855 | 1.090296 | 2.429316 | 1.780687 |
| chi-miR-211 | 2.49206968 | 1.294703 | 0.785669 | 1.635444 | 2.672248 | 2.828151 |
| chi-miR-21-3p | 0.276896631 | 0.863135 | 0.698372 | 1.635444 | 1.943453 | 1.361702 |
| chi-miR-214-3p | 165.7804498 | 119.9758 | 147.7057 | 201.2959 | 151.9537 | 241.545 |
| chi-miR-214-5p | 146.3398696 | 99.54825 | 176.2972 | 119.5237 | 87.45539 | 125.2766 |
| chi-miR-215-5p | 2.215173049 | 58.26162 | 101.875 | 1.771731 | 3.401043 | 2.932897 |
| chi-miR-21-5p | 25887.70845 | 21460.42 | 24381.74 | 41701.78 | 23847.14 | 41872.03 |
| chi-miR-216b | 89.16071524 | 0.719279 | 0.774593 | 0.136287 | 0.121466 | 0.418985 |
| chi-miR-217-5p | 80.43847135 | 1.870126 | 0.174593 | 0.545148 | 0.364397 | 0.104746 |
| chi-miR-218 | 3102.349856 | 573.4094 | 1006.32 | 498.4016 | 536.636 | 974.3502 |
| chi-miR-221-3p | 114.9150703 | 114.6531 | 97.07372 | 129.609 | 121.4658 | 110.1931 |
| chi-miR-221-5p | 58.28674086 | 35.96396 | 53.74465 | 46.88273 | 30.85232 | 43.36498 |
| chi-miR-222-3p | 77.94640167 | 62.72115 | 64.83131 | 54.65109 | 61.8261 | 48.81178 |
| chi-miR-223-3p | 0.353621365 | 0.5 | 0.611076 | 0.954009 | 0.485863 | 2.409165 |
| chi-miR-223-5p | 0.692241578 | 0.575423 | 0.611076 | 0.635444 | 0.121466 | 0.523732 |
| chi-miR-22-3p | 287.8340481 | 200.9666 | 100.7402 | 323.818 | 298.6844 | 200.0655 |
| chi-miR-224-3p | 0.138448316 | 0.287712 | 0.349186 | 0.272574 | 1.093192 | 1.047463 |
| chi-miR-224-5p | 661.2291552 | 322.2371 | 302.4406 | 328.8606 | 305.1221 | 293.9182 |
| chi-miR-22-5p | 8.381904694 | 14.09787 | 5.412384 | 26.84854 | 15.42616 | 13.09329 |
| chi-miR-2318 | 0.15 | 0.143856 | 0.174593 | 0.136287 | 0.214658 | 0.314239 |
| chi-miR-2331 | 0.692241578 | 0.287712 | 0.349186 | 0.545148 | 0.121466 | 0.209493 |
| chi-miR-2332 | 0.138448316 | 2.157838 | 0.523779 | 5.315194 | 1.579056 | 0.942717 |
| chi-miR-23a | 270.7592727 | 401.214 | 268.1749 | 367.5661 | 448.0874 | 439.9345 |
| chi-miR-23b-3p | 406.6612912 | 413.154 | 272.2778 | 448.7932 | 459.6266 | 470.6252 |
| chi-miR-23b-5p | 1.799828103 | 0.575423 | 0.749186 | 0.408861 | 0.485863 | 0.523732 |
| chi-miR-2404 | 0.138448316 | 0.1 | 0.087297 | 0.272574 | 0.2 | 0.104746 |
| chi-miR-2411-3p | 0.692241578 | 0.143856 | 0.436483 | 0.954009 | 0.485863 | 0.523732 |
| chi-miR-2411-5p | 3.738104521 | 1.438559 | 0.698372 | 1.908018 | 0.607329 | 1.466448 |
| chi-miR-24-3p | 5233.623226 | 1172.425 | 704.7448 | 1351.286 | 1052.258 | 1423.921 |
| chi-miR-24-5p | 193.6891935 | 26.75719 | 15.97526 | 21.26077 | 17.00521 | 31.10966 |
| chi-miR-2483-3p | 2.215173049 | 3.308685 | 1.047558 | 3.407175 | 1.943453 | 3.98036 |
| chi-miR-2483-5p | 1.661379787 | 4.459531 | 2.880785 | 6.132916 | 5.465962 | 5.551555 |
| chi-miR-25-3p | 1017.59512 | 993.4685 | 908.0657 | 856.155 | 913.0585 | 858.9198 |
| chi-miR-25-5p | 0.969138209 | 0.575423 | 0.349186 | 0.545148 | 0.728795 | 1.361702 |
| chi-miR-26a-3p | 2.076724734 | 1.150847 | 3.523779 | 1.36287 | 0.607329 | 2.723404 |
| chi-miR-26a-5p | 45517.17091 | 67529.1 | 42412.05 | 67011.92 | 83206.39 | 59256.67 |
| chi-miR-26b-3p | 0.276896631 | 0.863135 | 0.785669 | 0.954009 | 1.214658 | 0.104746 |
| chi-miR-26b-5p | 2371.933093 | 3262.219 | 2403.884 | 3237.498 | 4005.092 | 3496.432 |
| chi-miR-27a-3p | 342.8908451 | 572.5463 | 349.0988 | 501.9451 | 610.6087 | 553.3748 |
| chi-miR-27a-5p | 13.70638324 | 4.891099 | 9.76157 | 3.407175 | 4.251304 | 7.646481 |
| chi-miR-27b-3p | 4541.173421 | 6389.933 | 4230.302 | 6913.432 | 7161.746 | 6078.743 |
| chi-miR-27b-5p | 14.81396977 | 3.020973 | 3.317268 | 1.499157 | 2.429316 | 3.037643 |
| chi-miR-28-3p | 836.7816194 | 280.2312 | 347.1819 | 299.4226 | 252.8918 | 281.9771 |
| chi-miR-28-5p | 255.0217973 | 97.24656 | 101.8541 | 110.9376 | 95.83653 | 94.69067 |
| chi-miR-296-3p | 4.015001152 | 1.582414 | 2.095116 | 6.269203 | 3.158111 | 5.865794 |
| chi-miR-29a-3p | 1773.938268 | 1862.502 | 989.9425 | 1643.076 | 2304.935 | 1389.669 |
| chi-miR-29a-5p | 21.59793723 | 7.480504 | 15.0632 | 12.40212 | 5.465962 | 12.8838 |
| chi-miR-29b-3p | 848.9650712 | 421.9292 | 377.1246 | 323.1365 | 380.0665 | 293.9182 |
| chi-miR-29b-5p | 3.738104521 | 2.445549 | 1.436483 | 0.954009 | 1.093192 | 1.256956 |
| chi-miR-29c-5p | 165.1688405 | 39.4165 | 51.91143 | 48.92704 | 34.37483 | 40.53682 |
| chi-miR-301a-3p | 0.276896631 | 1.438559 | 0.611076 | 1.908018 | 1.943453 | 1.780687 |
| chi-miR-301a-5p | 0.553793262 | 0.301694 | 0.785669 | 3.543462 | 1.700521 | 1.361702 |
| chi-miR-301b | 0.138448316 | 0.519279 | 0.26189 | 1.499157 | 0.728795 | 1.152209 |
| chi-miR-30a-3p | 53.79592054 | 55.3845 | 43.12448 | 61.73802 | 59.63972 | 75.62684 |
| chi-miR-30a-5p | 3084.196512 | 4432.199 | 3325.124 | 4109.054 | 4481.36 | 5747.745 |
| chi-miR-30b-3p | 2.907414627 | 2.863135 | 1.222151 | 0.681435 | 2.064919 | 1.152209 |
| chi-miR-30b-5p | 224.6613 | 255.0564 | 198.7742 | 320.4108 | 391.2414 | 307.2209 |
| chi-miR-30c-3p | 1.384483156 | 1.150847 | 1.134855 | 1.36287 | 2.186385 | 2.094926 |
| chi-miR-30c-5p | 944.6328572 | 678.0759 | 594.9258 | 1116.054 | 740.9415 | 983.1489 |
| chi-miR-30d-3p | 4.860692197 | 4.17182 | 2.618895 | 6.269203 | 5.951825 | 4.399345 |
| chi-miR-30e-3p | 137.3985451 | 182.5531 | 116.9773 | 210.4272 | 156.5694 | 215.6727 |
| chi-miR-30e-5p | 952.5244112 | 898.8569 | 817.1827 | 1764.235 | 1869.237 | 1259.993 |
| chi-miR-30f-3p | 3.783967464 | 4.315676 | 1.920523 | 3.816037 | 4.372769 | 5.027823 |
| chi-miR-30f-5p | 180.1212586 | 62.5773 | 152.6398 | 75.6393 | 60.85437 | 91.86252 |
| chi-miR-320-3p | 589.0975828 | 522.916 | 630.812 | 514.7561 | 423.9157 | 548.2422 |
| chi-miR-323a-3p | 0.9 | 0.719279 | 1.020523 | 2.180592 | 1.336124 | 1.152209 |
| chi-miR-323b | 0.3 | 0.143856 | 0.436483 | 2.72574 | 0.485863 | 1.571195 |
| chi-miR-324-5p | 1.799828103 | 0.287712 | 0.785669 | 3.952324 | 2.550782 | 2.513912 |
| chi-miR-326-3p | 2.49206968 | 0.575423 | 0.436483 | 0.681435 | 0.364397 | 0.314239 |
| chi-miR-328-3p | 39.31932163 | 51.64425 | 33.78375 | 87.22369 | 74.33708 | 43.15548 |
| chi-miR-329a-3p | 0.2 | 0.287712 | 0.174593 | 0.408861 | 0.621466 | 0.837971 |
| chi-miR-329b-3p | 2.630517996 | 2.013982 | 5.586977 | 8.177221 | 3.886906 | 6.494272 |
| chi-miR-330-3p | 4.845691045 | 0.596396 | 2.357006 | 6.269203 | 2.793714 | 7.018003 |
| chi-miR-330-5p | 4.075865247 | 5.610378 | 3.142675 | 12.81098 | 4.980098 | 9.11293 |
| chi-miR-331-3p | 8.306898935 | 6.185802 | 2.793488 | 15.67301 | 13.36124 | 8.065466 |
| chi-miR-331-5p | 33.91983732 | 26.97095 | 12.22151 | 40.61353 | 31.45965 | 24.09165 |
| chi-miR-335-3p | 30.1817328 | 28.48346 | 37.28471 | 25.89453 | 19.67746 | 26.08183 |
| chi-miR-335-5p | 35.30432047 | 57.03683 | 21.38765 | 35.84349 | 66.56327 | 38.33715 |
| chi-miR-338-3p | 2.076724734 | 4.877117 | 2.00782 | 1.36287 | 3.76544 | 1.780687 |
| chi-miR-338-5p | 0.415344947 | 1.006991 | 0.698372 | 1.771731 | 2.672248 | 1.361702 |
| chi-miR-33a-3p | 1.038276418 | 0.575423 | 0.349186 | 1.36287 | 1.093192 | 1.99018 |
| chi-miR-33a-5p | 0.553793262 | 1.72627 | 0.698372 | 0.954009 | 1.45759 | 0.733224 |
| chi-miR-340-3p | 14.2601765 | 8.775207 | 15.23779 | 12.40212 | 3.886906 | 12.6743 |
| chi-miR-340-5p | 156.1697 | 117.5302 | 85.02681 | 189.7115 | 154.3831 | 190.8478 |
| chi-miR-342-3p | 398.0389073 | 361.9413 | 169.879 | 410.9054 | 363.9452 | 341.892 |
| chi-miR-342-5p | 0.830689893 | 1.006991 | 0.611076 | 1.36287 | 1.607329 | 1.047463 |
| chi-miR-3431-3p | 182.4748799 | 58.9809 | 39.10884 | 47.01902 | 38.50466 | 59.39116 |
| chi-miR-3431-5p | 253.7932623 | 325.5458 | 188.124 | 306.1006 | 391.8487 | 297.27 |
| chi-miR-3432-3p | 0.830689893 | 0.719279 | 0.785669 | 0.954009 | 0.971727 | 1.571195 |
| chi-miR-3432-5p | 80.93952578 | 81.71012 | 43.38637 | 106.3039 | 91.94962 | 90.81506 |
| chi-miR-345-3p | 3.230174201 | 6.185802 | 2.00782 | 5.315194 | 8.381141 | 4.294599 |
| chi-miR-345-5p | 0.384483156 | 0.719279 | 0.960262 | 1.226583 | 3.522509 | 1.047463 |
| chi-miR-34a | 45.41104751 | 32.22371 | 24.70491 | 67.32579 | 37.77587 | 36.13748 |
| chi-miR-34b-3p | 2.079613319 | 1.72627 | 1.134855 | 2.72574 | 3.643974 | 3.98036 |
| chi-miR-34c-3p | 0.475349554 | 0.143856 | 0.087297 | 0.6 | 0.607329 | 0.523732 |
| chi-miR-34c-5p | 12.16515444 | 13.66631 | 6.285349 | 21.66964 | 52.5947 | 23.25368 |
| chi-miR-361-3p | 456.8794414 | 183.7039 | 159.9272 | 267.9403 | 262.3998 | 253.4861 |
| chi-miR-361-5p | 44.4419093 | 49.34256 | 29.9427 | 50.4262 | 56.4816 | 54.67758 |
| chi-miR-362-3p | 0.938276418 | 1.150847 | 1.047558 | 0.954009 | 3.401043 | 1.99018 |
| chi-miR-362-5p | 25.05914512 | 33.2307 | 16.32445 | 32.84517 | 35.46802 | 30.79542 |
| chi-miR-363-3p | 1.799828103 | 2.445549 | 1.74593 | 1.635444 | 1.093192 | 1.618658 |
| chi-miR-365-3p | 18.35018342 | 17.11885 | 10.91206 | 21.80592 | 17.73401 | 14.87398 |
| chi-miR-369-3p | 6.645519148 | 13.09088 | 15.80067 | 32.02745 | 14.33297 | 26.29133 |
| chi-miR-369-5p | 1.107586525 | 1.294703 | 1.658634 | 6.814351 | 1.943453 | 3.666121 |
| chi-miR-374a-3p | 178.7367754 | 193.3423 | 133.5637 | 233.3234 | 211.2291 | 236.8314 |
| chi-miR-374a-5p | 411.3299456 | 492.994 | 304.0538 | 527.4308 | 609.2725 | 479.3191 |
| chi-miR-374b-3p | 15.36776303 | 14.38559 | 8.816948 | 16.62702 | 13.72564 | 17.59738 |
| chi-miR-374b-5p | 348.1975137 | 381.0742 | 276.5554 | 458.3332 | 677.5363 | 359.1751 |
| chi-miR-376a | 0.12 | 0.143856 | 0.087297 | 1.36287 | 0.121466 | 0.733224 |
| chi-miR-376b-3p | 0.692241578 | 1.870126 | 1.134855 | 4.770046 | 1.45759 | 3.770867 |
| chi-miR-376b-5p | 0.830689893 | 1.294703 | 0.872965 | 2.180592 | 1.728795 | 1.361702 |
| chi-miR-376c-3p | 2.353621365 | 3.164829 | 4.01564 | 3.952324 | 4.008372 | 6.913257 |
| chi-miR-376d | 0.453793262 | 0.143856 | 0.436483 | 0.545148 | 0.728795 | 0.523732 |
| chi-miR-376e-3p | 4.568794414 | 6.185802 | 5.848867 | 9.812665 | 6.437688 | 11.20786 |
| chi-miR-378-3p | 1686.992725 | 420.9222 | 958.6596 | 494.313 | 294.7975 | 806.8609 |
| chi-miR-378-5p | 1.922415779 | 3.596396 | 1.74593 | 2.72574 | 4.980098 | 2.513912 |
| chi-miR-379-3p | 6.230174201 | 6.617369 | 8.293169 | 13.76499 | 14.00837 | 17.91162 |
| chi-miR-379-5p | 218.7483386 | 214.7768 | 305.1013 | 690.0212 | 685.7212 | 699.4959 |
| chi-miR-380-3p | 10.93741693 | 8.343639 | 17.37201 | 32.30002 | 9.109936 | 26.71031 |
| chi-miR-381 | 19.5212125 | 47.47243 | 48.79875 | 95.5372 | 68.50466 | 111.4501 |
| chi-miR-382-3p | 7.753105673 | 10.35762 | 20.95116 | 33.25403 | 13.23977 | 30.79542 |
| chi-miR-382-5p | 9.41448546 | 8.631351 | 12.30881 | 19.21647 | 15.83036 | 25.5581 |
| chi-miR-383 | 1.107586525 | 4.241729 | 4.190233 | 5.315194 | 11.29632 | 9.636661 |
| chi-miR-3955-3p | 0.18 | 0.287712 | 0.087297 | 0.136287 | 0.364397 | 0.25 |
| chi-miR-3955-5p | 1.384483156 | 1.082414 | 1.309448 | 2.862027 | 1.45759 | 2.618658 |
| chi-miR-3958-3p | 26.84569105 | 26.61333 | 26.97462 | 104.3959 | 30.00206 | 60.85761 |
| chi-miR-3959-3p | 2.076724734 | 5.178811 | 6.459942 | 11.85697 | 5.587428 | 13.82651 |
| chi-miR-3959-5p | 30.32018111 | 54.0898 | 74.55122 | 168.0419 | 56.23867 | 118.1538 |
| chi-miR-409-3p | 7.19931241 | 19.5644 | 27.14922 | 75.91187 | 14.9403 | 65.88543 |
| chi-miR-409-5p | 5.122587677 | 11.22076 | 17.6339 | 38.02408 | 39.9602 | 48.70704 |
| chi-miR-410-3p | 1.4 | 1.150847 | 1.74593 | 8.449795 | 4.700521 | 4.608838 |
| chi-miR-411a-3p | 2.215173049 | 4.315676 | 7.071018 | 8.994943 | 4.372769 | 13.09329 |
| chi-miR-411a-5p | 109.3741693 | 200.6789 | 228.8915 | 426.9872 | 217.6667 | 365.7741 |
| chi-miR-411b-5p | 1.384483156 | 4.17182 | 3.75375 | 8.722369 | 5.587428 | 5.446809 |
| chi-miR-412-5p | 0.415344947 | 3.308685 | 4.714012 | 18.26246 | 12.42932 | 13.19804 |
| chi-miR-421-3p | 2.614657357 | 3.596396 | 1.74593 | 4.224898 | 4.737167 | 3.875614 |
| chi-miR-423-3p | 88.33002534 | 161.1186 | 69.74992 | 272.1652 | 240.1379 | 131.8756 |
| chi-miR-423-5p | 207.8340481 | 174.7849 | 107.6366 | 265.6234 | 192.2804 | 247.0966 |
| chi-miR-424-3p | 2.907414627 | 32.65528 | 16.58634 | 40.88611 | 46.60101 | 41.68903 |
| chi-miR-424-5p | 78.58379557 | 73.49016 | 92.53431 | 168.4508 | 168.8375 | 229.3944 |
| chi-miR-425-3p | 3.876552836 | 1.582414 | 2.222151 | 2.862027 | 0.971727 | 1.466448 |
| chi-miR-425-5p | 131.5258998 | 48.33557 | 94.74401 | 69.77895 | 38.26173 | 86.93944 |
| chi-miR-429 | 27.5512148 | 3.870126 | 2.095116 | 0.545148 | 2.186385 | 0.628478 |
| chi-miR-432-5p | 1.938276418 | 8.631351 | 8.642355 | 18.80761 | 16.07329 | 25.76759 |
| chi-miR-433 | 0.415344947 | 0.863135 | 1.396744 | 4.224898 | 1.214658 | 3.561375 |
| chi-miR-449a-5p | 28.79724964 | 3.582414 | 0.611076 | 0.545148 | 2.793714 | 0.418985 |
| chi-miR-450-3p | 0.276896631 | 1.438559 | 0.174593 | 2.589453 | 2.242932 | 1.466448 |
| chi-miR-450-5p | 20.35190239 | 192.0274 | 120.5565 | 402.7281 | 239.6521 | 340.3208 |
| chi-miR-451-5p | 339.0599249 | 223.9836 | 164.4666 | 256.6285 | 206.3704 | 111.2995 |
| chi-miR-454-3p | 7.060864095 | 9.35063 | 5.586977 | 13.49241 | 12.31622 | 11.94108 |
| chi-miR-455-3p | 26.99742154 | 105.0148 | 71.67044 | 148.9617 | 114.0564 | 98.04255 |
| chi-miR-455-5p | 671.4743306 | 2695.311 | 2260.543 | 4600.777 | 3378.45 | 3245.146 |
| chi-miR-483 | 0.276896631 | 0.431568 | 1.047558 | 1.635444 | 1.579056 | 2.513912 |
| chi-miR-485-3p | 0.8 | 0.719279 | 0.960262 | 1.090296 | 0.728795 | 1.047463 |
| chi-miR-485-5p | 0.830689893 | 1.438559 | 2.269709 | 3.543462 | 2.186385 | 3.037643 |
| chi-miR-487a-3p | 0.276896631 | 0.287712 | 1.134855 | 1.090296 | 0.971727 | 1.466448 |
| chi-miR-487b-3p | 3.045862943 | 4.603387 | 5.586977 | 11.31182 | 4.737167 | 8.798691 |
| chi-miR-490 | 15.97898244 | 11.79618 | 15.27689 | 17.44474 | 18.09841 | 19.16858 |
| chi-miR-491-5p | 3.045862943 | 2.877117 | 1.658634 | 5.724055 | 4.251304 | 5.13257 |
| chi-miR-493-3p | 29.3510429 | 48.19171 | 73.06718 | 169.9499 | 53.68583 | 173.2504 |
| chi-miR-493-5p | 8.722243882 | 37.40252 | 45.21959 | 136.287 | 121.8638 | 88.51064 |
| chi-miR-494 | 48.0300023 | 45.45845 | 52.98898 | 34.75367 | 35.10362 | 94.37643 |
| chi-miR-495-3p | 9.41448546 | 16.25571 | 20.34009 | 49.06333 | 18.94867 | 35.509 |
| chi-miR-497-5p | 102.0364086 | 145.4383 | 83.36817 | 133.0161 | 183.7778 | 142.9787 |
| chi-miR-499-5p | 38.35018342 | 42.72115 | 41.64044 | 67.46207 | 55.14548 | 60.12439 |
| chi-miR-500-3p | 15.22931471 | 24.02393 | 14.57852 | 31.61859 | 32.30991 | 27.65303 |
| chi-miR-500-5p | 8.999140513 | 12.65931 | 9.951803 | 14.99157 | 13.11831 | 14.76923 |
| chi-miR-502b-3p | 0.138448316 | 0.287712 | 0.26189 | 0.681435 | 0.242932 | 0.209493 |
| chi-miR-502b-5p | 0.830689893 | 1.294703 | 0.349186 | 2.316879 | 1.45759 | 1.571195 |
| chi-miR-504 | 12.87569335 | 8.343639 | 6.198053 | 14.99157 | 9.437688 | 8.484452 |
| chi-miR-505-3p | 11.07586525 | 11.2627 | 8.380465 | 18.12617 | 14.9403 | 13.51227 |
| chi-miR-532-3p | 6.645519148 | 9.926054 | 7.682093 | 9.812665 | 13.48271 | 11.62684 |
| chi-miR-532-5p | 556.5622286 | 781.2811 | 447.6565 | 810.7715 | 921.3182 | 779.3126 |
| chi-miR-542-3p | 63.68622517 | 300.7945 | 235.6133 | 521.4341 | 334.5169 | 717.8265 |
| chi-miR-542-5p | 0.138448316 | 1.027964 | 1.396744 | 3.816037 | 2.550782 | 4.399345 |
| chi-miR-543-3p | 2.353621365 | 4.459531 | 6.634535 | 13.90128 | 4.615701 | 14.2455 |
| chi-miR-544-5p | 0.138448316 | 1.006991 | 1.047558 | 1.090296 | 1.336124 | 0.733224 |
| chi-miR-545-3p | 0.3 | 0.287712 | 0.349186 | 0.408861 | 0.728795 | 0.704746 |
| chi-miR-582-5p | 0.184311258 | 0.575423 | 0.960262 | 1.771731 | 1.700521 | 1.047463 |
| chi-miR-592 | 2.225017278 | 2.877117 | 6.110756 | 2.044305 | 4.494235 | 9.427169 |
| chi-miR-628-5p | 0.768966312 | 1.294703 | 0.960262 | 0.817722 | 1.336124 | 1.047463 |
| chi-miR-655 | 0.830689893 | 2.733261 | 4.01564 | 8.586082 | 5.344496 | 7.751227 |
| chi-miR-656 | 0.19 | 0.287712 | 0.087297 | 0.545148 | 0.485863 | 1.047463 |
| chi-miR-660 | 48.73380709 | 58.40548 | 30.99026 | 85.0431 | 71.4219 | 53.63011 |
| chi-miR-671-3p | 1.938276418 | 1.72627 | 1.047558 | 2.862027 | 2.728795 | 3.247136 |
| chi-miR-671-5p | 4.015001152 | 5.034955 | 3.491861 | 3.952324 | 3.522509 | 6.389525 |
| chi-miR-708-3p | 191.7509171 | 207.9954 | 173.0217 | 310.4618 | 247.9117 | 329.4272 |
| chi-miR-708-5p | 17.72138439 | 43.44447 | 19.90361 | 33.52661 | 47.6146 | 39.59411 |
| chi-miR-7-3p | 33.22759574 | 14.96101 | 17.76939 | 10.76667 | 11.53925 | 16.9689 |
| chi-miR-758 | 0.138448316 | 3.020973 | 2.793488 | 7.359499 | 5.943453 | 5.027823 |
| chi-miR-7-5p | 1092.080313 | 1280.893 | 1497.59 | 996.2581 | 641.8254 | 1051.025 |
| chi-miR-767 | 4.153449467 | 4.466522 | 2.880785 | 8.313508 | 4.858633 | 2.723404 |
| chi-miR-874-3p | 2.49206968 | 3.740252 | 2.444302 | 7.632073 | 10.20313 | 4.713584 |
| chi-miR-874-5p | 0.276896631 | 0.143856 | 0.087297 | 0.545148 | 1.093192 | 0.104746 |
| chi-miR-877-5p | 4.153449467 | 1.006991 | 1.047558 | 4.361185 | 1.093192 | 5.237316 |
| chi-miR-92a-3p | 199.0886778 | 489.5415 | 228.7169 | 569.4072 | 655.6725 | 403.3781 |
| chi-miR-92a-5p | 0.138448316 | 0.2 | 0.26189 | 0.408861 | 0.56 | 0.733224 |
| chi-miR-93-3p | 0.272574037 | 0.287712 | 0.26189 | 0.83069 | 0.850261 | 0.837971 |
| chi-miR-93-5p | 205.0419554 | 202.9725 | 104.4939 | 241.6369 | 233.8217 | 207.7119 |
| chi-miR-9-3p | 4.845691045 | 0.719279 | 0.872965 | 0.408861 | 1.579056 | 0.837971 |
| chi-miR-9-5p | 259.8674883 | 48.91099 | 49.46712 | 38.97809 | 41.78424 | 35.40426 |
| chi-miR-96 | 0.352761878 | 0.863135 | 0.611076 | 0.136287 | 1.45759 | 0.942717 |
| chi-miR-98-5p | 456.1871998 | 340.6507 | 289.2588 | 452.7455 | 279.7358 | 282.7103 |
| chi-miR-99a-3p | 23.25931702 | 97.39041 | 51.36945 | 77.81989 | 150.8605 | 59.07692 |
| chi-miR-99a-5p | 142908.1512 | 172543.3 | 172600.7 | 142348 | 153894.2 | 153436.9 |
| chi-miR-99b-3p | 45.41104751 | 16.11186 | 32.04692 | 26.16711 | 14.2115 | 23.46318 |
| chi-miR-99b-5p | 995.1664924 | 956.4976 | 942.1077 | 930.0226 | 837.3853 | 926.2717 |
| novel_110 | 10.6605203 | 6.617369 | 3.666454 | 7.086925 | 3.76544 | 4.504092 |
| novel_143 | 3.553793262 | 3.466522 | 3.317268 | 10.90296 | 4.008372 | 7.12275 |
| novel_160 | 3.876552836 | 3.020973 | 5.74593 | 0.817722 | 1.943453 | 4.923077 |
| novel_167 | 1.522931471 | 3.020973 | 2.444302 | 0.817722 | 1.093192 | 1.932897 |
| novel_183 | 0.138448316 | 0.719279 | 0.611076 | 0.954009 | 1.121466 | 1.361702 |
| novel_185 | 1.384483156 | 0.719279 | 0.936483 | 0.272574 | 0.607329 | 0.523732 |
| novel_201 | 0.830689893 | 0.431568 | 0.087297 | 0.545148 | 0.242932 | 0.209493 |
| novel_212 | 0.1 | 0.143856 | 0.087297 | 0.553793 | 0.485863 | 0.104746 |
| novel_215 | 0.138448316 | 0.087712 | 0.087297 | 0.954009 | 0.242932 | 0.209493 |
| novel_227 | 0.138448316 | 0.143856 | 0.13 | 0.136287 | 0.2 | 0.314239 |
| novel_231 | 0.138448316 | 0.143856 | 0.13 | 0.272574 | 0.221466 | 0.25 |
| novel_233 | 0.08 | 0.087712 | 0.087297 | 0.545148 | 0.242932 | 0.209493 |
| novel_255 | 0.276896631 | 0.287712 | 0.27 | 0.272574 | 0.4 | 0.523732 |
| novel_261 | 0.215173049 | 0.287712 | 0.26189 | 0.954009 | 0.485863 | 1.047463 |
| novel_266 | 1.24603484 | 0.431568 | 1.698372 | 0.545148 | 0.8 | 1.047463 |
| novel_270 | 7.337760726 | 2.445549 | 6.571337 | 4.361185 | 1.700521 | 5.656301 |
| novel_272 | 0.138448316 | 0.15 | 0.174593 | 0.545148 | 0.35 | 0.209493 |
| novel_285 | 0.138448316 | 0.287712 | 0.174593 | 0.32 | 0.342932 | 0.314239 |
| novel_296 | 0.415344947 | 0.287712 | 0.26189 | 1.226583 | 0.485863 | 0.733224 |
| novel_308 | 0.272574037 | 0.143856 | 0.2 | 5.537933 | 3.76544 | 10.57938 |
| novel_316 | 0.415344947 | 0.431568 | 0.960262 | 0.681435 | 0.485863 | 1.361702 |
| novel_319 | 0.25 | 1.164829 | 0.349186 | 1.771731 | 2.064919 | 0.523732 |
| novel_333 | 0.692241578 | 0.485863 | 0.698372 | 1.36287 | 1.150847 | 2.094926 |
| novel_359 | 0.2 | 0.143856 | 0.174593 | 0.408861 | 0.221466 | 0.314239 |
| novel_360 | 0.408861055 | 0.575423 | 0.26189 | 0.969138 | 0.364397 | 0.733224 |
| novel_363 | 0.692241578 | 0.143856 | 0.436483 | 1.36287 | 0.364397 | 0.942717 |
| novel_370 | 0.138448316 | 0.15 | 0.174593 | 0.681435 | 0.121466 | 0.523732 |
| novel_378 | 0.4 | 0.431568 | 0.387297 | 0.136287 | 0.121466 | 0.209493 |
| novel_399 | 0.692241578 | 0.575423 | 0.587297 | 0.272574 | 0.121466 | 0.209493 |
| novel_42 | 140.9000691 | 168.4552 | 136.3572 | 233.8685 | 257.9934 | 273.8069 |
| novel_427 | 0.138448316 | 0.575423 | 0.35 | 0.136287 | 0.621466 | 0.209493 |
| novel_428 | 2.353621365 | 4.17182 | 2.269709 | 4.906333 | 4.251304 | 10.05565 |
| novel_435 | 14.95241808 | 6.617369 | 13.40456 | 10.22153 | 3.76544 | 10.47463 |
| novel_438 | 0.692241578 | 0.56 | 0.487297 | 0.408861 | 0.35 | 0.314239 |
| novel_449 | 0.276896631 | 0.7 | 1.134855 | 1.090296 | 1.214658 | 1.256956 |
| novel_451 | 0.138448316 | 0.287712 | 0.087297 | 0.136287 | 0.121466 | 0.314239 |
| novel_454 | 0.276896631 | 0.431568 | 0.35 | 0.136287 | 0.364397 | 0.25 |
| novel_456 | 0.692241578 | 0.143856 | 0.837971 | 0.136287 | 0.1 | 0.087297 |
| novel_462 | 0.969138209 | 1.72627 | 1.611076 | 0.408861 | 1.45759 | 1.152209 |
| novel_472 | 0.15 | 0.143856 | 0.174593 | 0.28 | 0.242932 | 0.304746 |
| novel_473 | 0.830689893 | 0.431568 | 0.487297 | 0.545148 | 0.364397 | 0.314239 |
| novel_483 | 0.415344947 | 0.287712 | 0.474593 | 0.272574 | 0.242932 | 0.314239 |
| novel_52 | 62.30174201 | 70.34551 | 51.59224 | 81.9085 | 88.42711 | 74.89362 |
| novel_63 | 22.69224158 | 97.24656 | 27.4984 | 77.6836 | 102.8815 | 116.0589 |
| novel_88 | 23.1208687 | 20.42349 | 14.22933 | 38.29665 | 38.0188 | 28.59574 |
| novel_94 | 22.29017881 | 21.72223 | 10.0391 | 26.71226 | 34.73922 | 27.33879 |
| novel_96 | 17.94640167 | 18.05593 | 17.15831 | 10.35781 | 11.53925 | 9.9509 |
| novel_98 | 4.153449467 | 4.315676 | 4.452122 | 16.7633 | 19.9801 | 21.68249 |
